# Natural variation of a sensor kinase controlling a conserved stress response pathway in *Escherichia coli*

**DOI:** 10.1371/journal.pgen.1007101

**Published:** 2017-11-15

**Authors:** Manuela Roggiani, Srujana S. Yadavalli, Mark Goulian

**Affiliations:** Department of Biology, University of Pennsylvania, Philadelphia, PA, United States of America; University of Geneva Medical School, SWITZERLAND

## Abstract

Previous studies have shown that exponentially growing *Escherichia coli* can detect mild acidity (~pH 5.5) and, in response, synthesize enzymes that protect against severe acid shock. This adaptation is controlled by the EvgS/EvgA phosphorelay, a signal transduction system present in virtually every *E*. *coli* isolate whose genome has been sequenced. Here we show that, despite this high level of conservation, the EvgS/EvgA system displays a surprising natural variation in pH-sensing capacity, with some strains entirely non-responsive to low pH stimulus. In most cases that we have tested, however, activation of the EvgA regulon still confers acid resistance. From analyzing selected *E*. *coli* isolates, we find that the natural variation results from polymorphisms in the sensor kinase EvgS. We further show that this variation affects the pH response of a second kinase, PhoQ, which senses pH differently from the closely related PhoQ in *Salmonella enterica*. The within-species diversification described here suggests EvgS likely responds to additional input signals that may be correlated with acid stress. In addition, this work highlights the fact that even for highly conserved sensor kinases, the activities identified from a subset of isolates may not necessarily generalize to other members of the same bacterial species.

## Author summary

Bacteria employ a class of proteins, sensor kinases, to sense environmental cues and initiate cellular responses through phosphorylation of partner response regulator proteins. Individual kinases are generally assumed to have the same sensory activity across members of a bacterial species. In this work, we report an unexpected counterexample in which the well-established capacity of the kinase EvgS to sense mild acidity is limited to a subset of *Escherichia coli* isolates. Despite this natural variation, EvgS activation still confers resistance to acid stress in strains that have lost EvgS pH-sensing activity. Thus, most *E*. *coli* share a conserved output of the Evg system but do not require identical sensory functions. This work highlights the potential for significant functional divergence of a sensor kinase within a species and also indicates that there are additional input signals for the highly conserved EvgS protein.

## Introduction

The species *Escherichia coli* comprises a remarkably diverse collection of bacteria, reflecting their capacity to colonize and manipulate disparate *in vivo* and *ex vivo* niches. Most of the well-documented phenotypic differences between *E*. *coli* strains are associated with genes found in only a subset of isolates [1]. Genes that are ubiquitous or nearly ubiquitous across the species, on the other hand, are generally assumed to have the same function in each cell type. However, polymorphisms in these core genes could have significant effects on the activities of the proteins that they encode and contribute to natural variation across the species. Furthermore, highly conserved networks of interacting proteins can be perturbed by components that are not encoded in all strains. Thus, the properties of a conserved regulatory circuit may depend on the *E*. *coli* isolate and be quite different from the properties established in the well-studied laboratory strain, *E*. *coli* K-12. Here we report an unexpected example of such natural variation in the EvgS/EvgA phosphorelay, a two-component system that has been identified in virtually all *E*. *coli* isolates.

**Fig 1:**
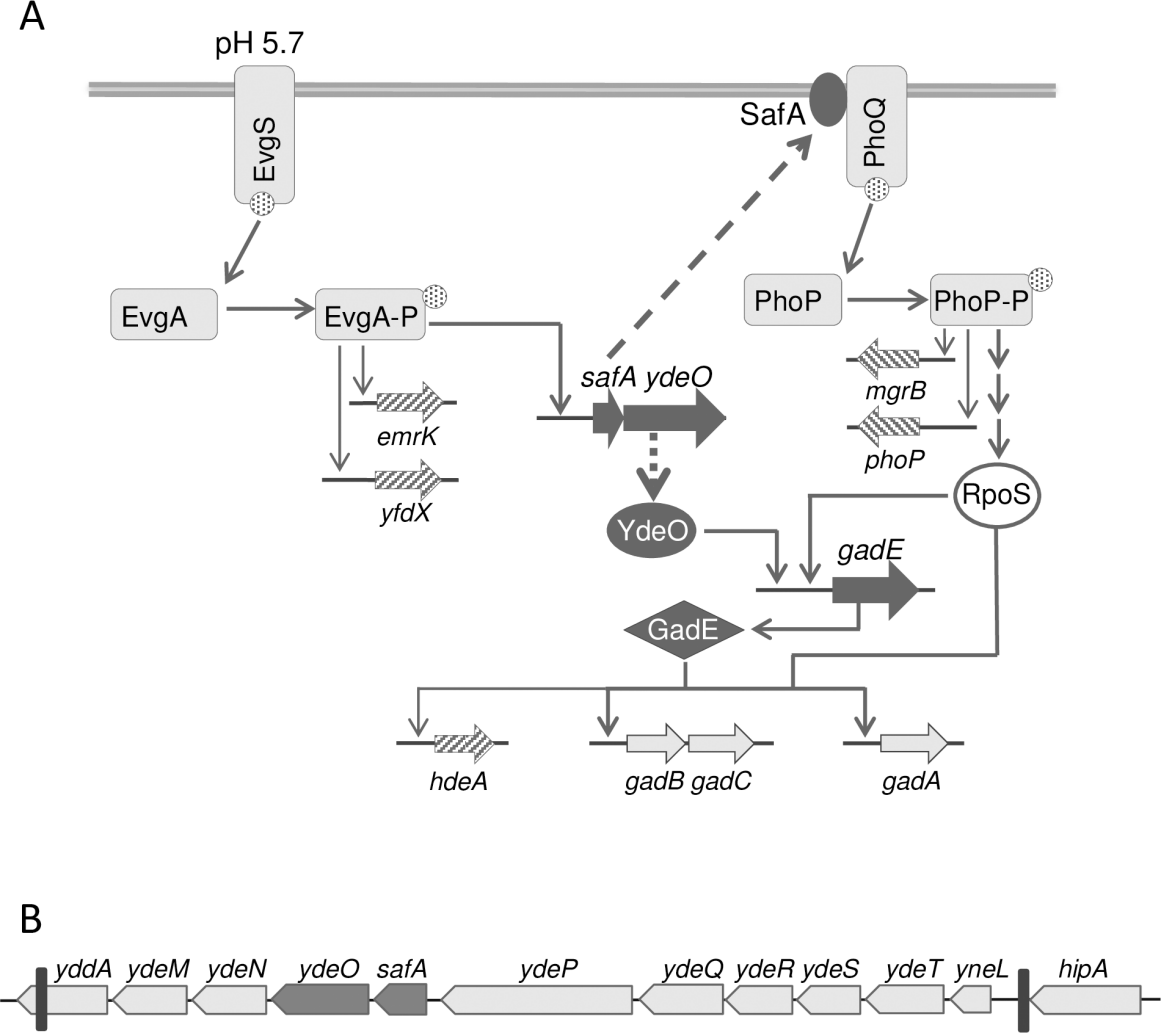
The Evg acid resistance network. A: Simplified diagram of the regulatory pathway leading to acid resistance in exponential phase-minimal medium cultures of *E*. *coli*. Thick arrows represent genes, with black denoting regulators, and hatched arrows indicate genes whose promoters were used as reporters in this study. Gray rectangles represent two-component system proteins, black ovals and diamonds represent other types of regulators. For clarity, only representative AR effector-encoding genes are shown, and genes of the PhoP regulon are omitted. B: Locus in MG1655 containing *safA* and *ydeO* (depicted as dark grey arrows) that is missing in *E. coli* MP1. Black vertical lines represent the boundaries of the region missing in MP1. Figure not drawn to scale.

The *E*. *coli* EvgS/EvgA phosphorelay is at the top of a pathway associated with acid and drug resistance [2–10]. Studies of this system in *E*. *coli* K-12 indicate that the sensor kinase EvgS is stimulated by mild acidity (pH 5.5–5.7), possibly via the protein’s periplasmic domain [9–11], resulting in EvgS autophosphorylation and subsequent phosphoryl transfer to the response regulator EvgA [12]. Phosphorylated EvgA regulates transcription of a number of genes, including the *safAydeO* operon, which is a node for two branches of the Evg network (Fig 1A) [6]. The transcription factor YdeO is a key component of the glutamate-dependent acid resistance network AR2 [2, 7, 8, 13] (for comprehensive reviews on *E*. *coli* acid resistance (AR) systems see [14–16]) that upregulates the activator GadE, leading to increased expression of AR2 effector genes. SafA encodes a small membrane protein that activates the sensor kinase PhoQ, thereby connecting the EvgS/EvgA and PhoQ/PhoP two-component signaling systems (Fig 1A) [17, 18]. PhoQ is stimulated by conditions of low divalent cations (Mg^++^ and Ca^++^) and antimicrobial peptides [19, 20]. In Salmonella, PhoQ is also stimulated directly by low pH [21], but in *E*. *coli* pH stimulation of PhoQ is indirect via SafA [8]. PhoQ controls the phosphorylation state of the response regulator PhoP, which in turn regulates transcription of a large regulon that includes genes associated with acid resistance. In exponential phase cells, PhoP contributes to AR2 by indirectly elevating RpoS levels [22], which contributes to the expression of the central regulator GadE as well as downstream effectors (Fig 1A) [22]. Thus, EvgS is believed to contribute to acid resistance via both the SafA and YdeO branches of the pathway.

The *evgAevgS* operon is found in almost all of the fully-sequenced *E*. *coli* genomes currently in the NCBI database. In contrast with this almost universal conservation among *E*. *coli*, close orthologs of *evgA* and *evgS* have not been identified in other bacterial species, including other species within the *Escherichia* genus. In addition, the *safAydeO* operon has a similarly high level of conservation in *E*. *coli* and, like *evgAevgS*, has not been identified in other species.

Recently, we noticed that *safAydeO* is missing in *E*. *coli* MP1, a mouse commensal isolate [23], suggesting key links in the Evg network may be severed in this strain. This observation was the starting point for the work presented here. While establishing the effects of these missing genes, we determined that EvgS is unresponsive to pH in MP1, as well as in many other *E*. *coli* isolates, despite the high conservation of the Evg network. We also show that the divergence is due to natural variation in the EvgS sequence. In addition, we find that low pH activation of the PhoQ/PhoP system is similarly variable across *E*. *coli*.

## Results

### *E*. *coli* MP1 lacks two branches of the EvgS/EvgA pathway

*E*.*coli* strain MP1 lacks a 13 kb segment of DNA containing the genes *safA* and *ydeO* that mediate two branches of the EvgS/EvgA AR2 pathway (Fig 1A and 1B). To explore the physiological effects of this disruption on the Evg network, we monitored the transcription of a PhoQ/PhoP-regulated promoter (P_*mgrB*_) [24], [25] and a GadE-regulated promoter (P_*hdeA*_) [6, 26] using fluorescent protein fusions. The reporter constructs were integrated in the chromosome at ectopic sites, leaving the native loci undisturbed.

**Fig 2:**
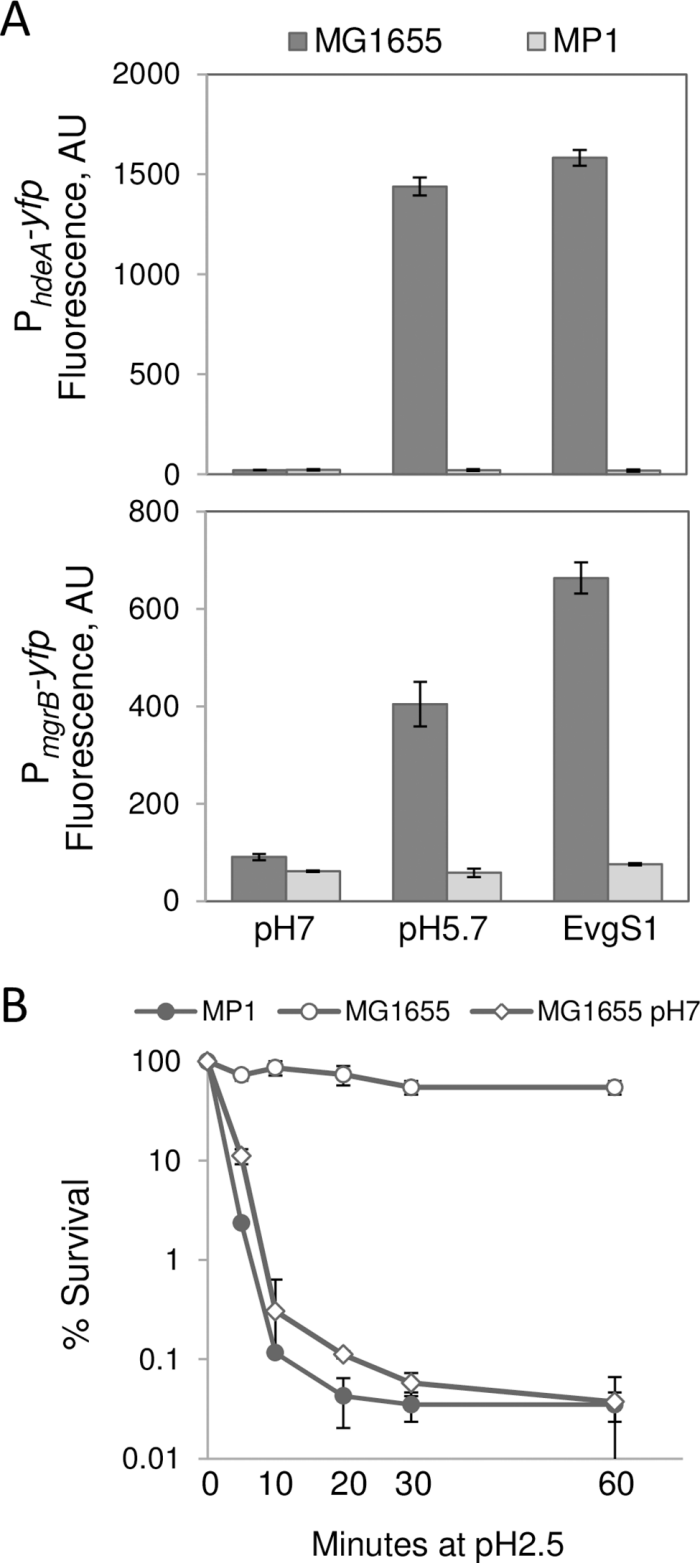
Two branches of the EvgS/EvgA pathway are interrupted, and acid resistance in exponential phase cultures is severely impaired in MP1. A: Activation of fluorescent reporters. Strains MMR175, MMR180, MP138, MP139 (top panel, P_*hdeA*_*-yfp* reporter) and strains TIM63, MMR170, MP131, and MP140 (bottom panel, P_*mgrB*_*-yfp* reporter) were grown in minimal medium at pH 5.7 (induced wild-type strains) and pH 7 (*evgS1* strains and non-induced wild-type strains) to OD_600_ ~0.2. Cultures of the P_*mgrB*_*-yfp* reporter strains contained 10 mM Mg^++^ so that PhoQ activation was fairly low in the absence of pH induction. Fluorescence was quantified as described in Materials and methods. Values represent the average fluorescence from two representative experiments and error bars represent the range. B: Survival after acid challenge at pH 2.5 for various times. Strains MG1655 and MP1 were cultured in minimal medium at pH 5.7 and pH 7 (MG1655 only) to OD_600_ ~0.2. Cultures were shocked in LB at pH 2.5, and aliquots were withdrawn at the indicated times. Values are the average percent survival from two representative experiments and error bars represent the range.

The EvgS/EvgA two-component system is activated by mild acidity (pH 5.5–5.7) in glucose minimal medium [7, 8, 10]. In addition, the signaling cascade can be initiated with a constitutively active EvgS variant, EvgS1, that has the amino acid substitution F577S [4, 6, 22]. Both mild acidity and the presence of the EvgS1 allele activated *mgrB* and *hdeA* transcription in the standard laboratory strain *E*. *coli* K-12 (MG1655). However, neither condition had an effect on transcription of these genes in *E*. *coli* MP1 (Fig 2A). Transcription from the *mgrB* promoter in MP1 was not stimulated over a range of acid pH values (5.1–7) and was similar to the behavior of MG1655 *ΔsafA* (S1A Fig). To rule out the possibility that the PhoQ/PhoP system itself was compromised in MP1, we verified that *mgrB* transcription is activated by low Mg^2+^ in a similar fashion in both MG1655 and MP1 (S1B Fig). These results are consistent with the observation that *safA* and *ydeO* are absent in the MP1 genome and that no other proteins in MP1 perform equivalent functions.

The absence of two branches of the Evg network suggests that MP1 may be less acid resistant than other *E*. *coli* isolates. We therefore grew MP1 to exponential phase in minimal medium at pH 5.7, to induce the Evg system, and then shocked the cells at pH 2.5 in rich medium, as described previously [13]. After one hour, MP1 survival was over 3 orders of magnitude lower than the survival of MG1655 (Fig 2B). This difference emerged quite rapidly, with MP1 showing a 40-fold reduction compared to MG1655 by 5 minutes following the transition to pH 2.5. The acid shock sensitivity of MP1 in these growth conditions was comparable to that of MG1655 cultures in exponential phase at neutral pH, a condition for which the EvgS/EvgA two-component system is inactive (Fig 2B) [2, 7, 8, 13, 27]. In contrast, MP1 and MG1655 in stationary phase withstand acid challenge equally well, regardless of pre-exposure to mildly acidic pH (S2A Fig). These observations confirm that inducible exponential phase acid resistance is impaired in MP1, consistent with the absence of *safAydeO*.

### Restoring *safAydeO* in MP1 is not sufficient for low pH activation of GadE and PhoQ

**Fig 3:**
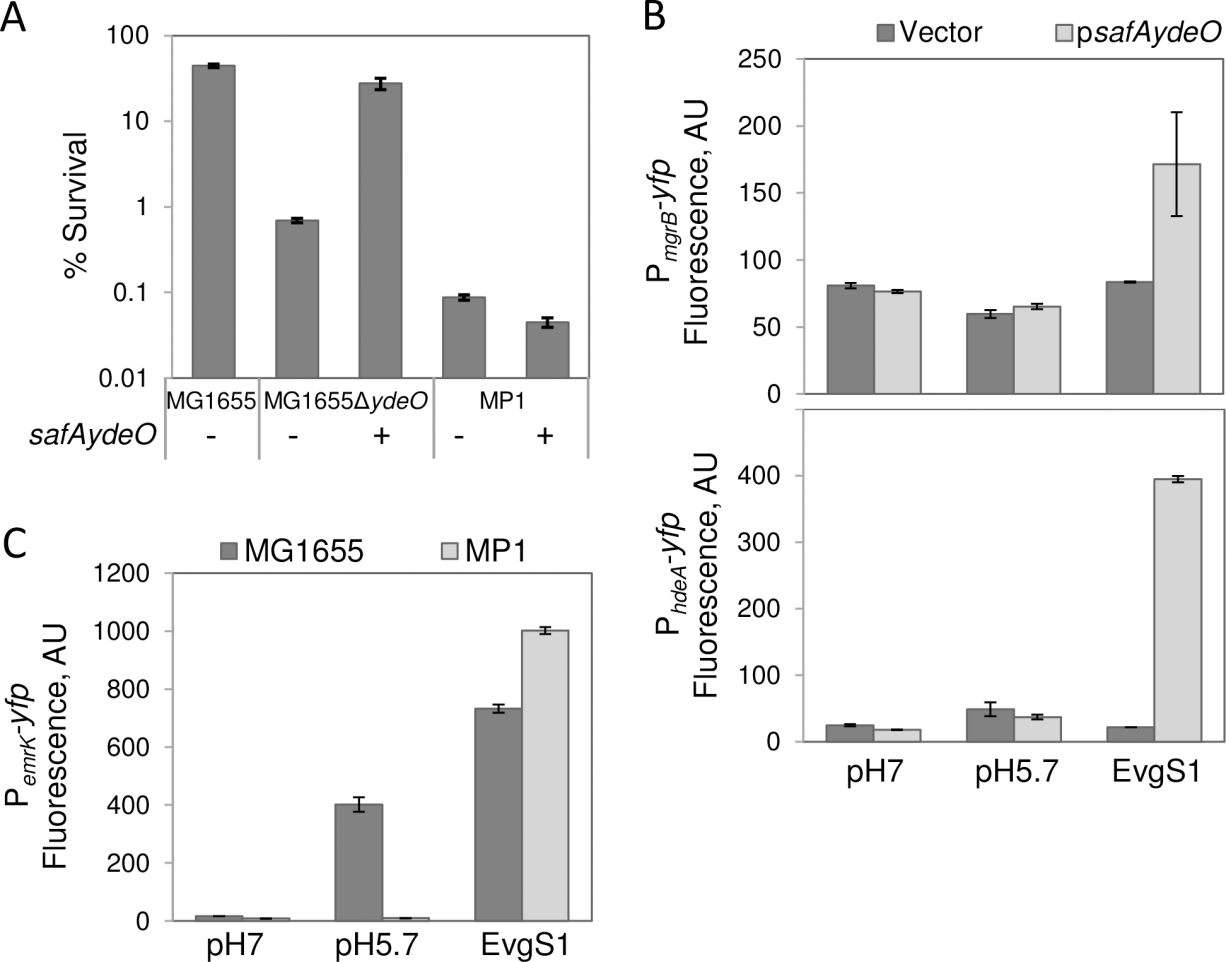
EvgS does not respond to mild pH induction in MP1. A: Resistance to acid challenge. Strains MG1655 or its Δ*ydeO* derivative (MMR239), and MP1, with the empty vector pSMART or plasmid p*safAydeO*_MG1655_ (pMR76) were cultured in minimal medium at pH 5.7 to OD_600_ ~0.2, and shocked for an hour at pH 2.5 in LB as described in Materials and methods. Percent survival values are the average of three independent experiments and error bars represent the standard deviations of the means. B: Activation of fluorescent reporters. Strains MP142 and MP143 (top panel, P_*mgrB*_*-yfp* reporter) and strains MP138 and MP139 (bottom panel, P_*hdeA*_*-yfp* reporter), each carrying the empty vector pSMART or its derivative p*safAydeO*_MG1655_ (pMR76) were cultured in minimal medium at pH 5.7 (induced wild-type strains) and pH 7 (*evgS1* strains and non-induced wild type strains) to OD_600_ ~0.2 and fluorescence was quantified as described in Materials and methods. Fluorescence values are the average of two representative experiments and error bars represent the range. C: As in (B), but with strains MMR173, MMR179, MP136, and MP137 (P_*emrK*_*-yfp* reporter).

To determine whether the *safAydeO* operon from MG1655 (Fig 1B) would restore the interrupted EvgS-PhoQ and EvgS-GadE pathways in MP1, we introduced this segment of DNA into MP1 on a single copy plasmid (p*safAydeO*_MG1655_), see Materials and methods. Comparison of the acid resistance of MP1 carrying either the empty vector or p*safAydeO*_MG1655_ revealed that restoration of *safAydeO* did not increase survival of MP1 (Fig 3A). However, p*safAydeO* does complement a *ydeO* deletion in MG1655 and fully restores acid resistance in this strain. In addition, transduction of a segment of DNA that includes the full 13 kb region missing in MP1 (Fig 1B) failed to rescue acid resistance (S2B Fig).

We also found that MP1/ p*safAydeO*_MG1655_ did not activate transcription of the PhoQ/PhoP and GadE reporters P_*mgrB*_*-yfp* and P_*hdeA*_*-yfp*, respectively, in response to low pH (Fig 3B). In contrast, the constitutively active EvgS_MG1655_ variant EvgS1 activated expression of both reporters when p*safAydeO*_MG1655_ was present. These results indicate that the SafA-PhoQ and YdeO-GadE interactions were successfully restored by p*safAydeO*_MG1655_ in MP1 and that components upstream of *safA* and *ydeO* involved in pH sensing are divergent between MP1 and MG1655.

It is also noteworthy that in MG1655, neither *safA* nor *phoQP* deletions affected acid resistance, even after prolonged exposure to low pH, in contrast with the behavior of a Δ*evgAS* strain (S2C Fig). These results indicate that for exponential phase cultures, the SafA-PhoQ-PhoP branch of the Evg network does not provide increased protection to acid shock following induction at pH 5.7.

### The MP1 EvgS/EvgA system is not induced by low pH

**Fig 4:**
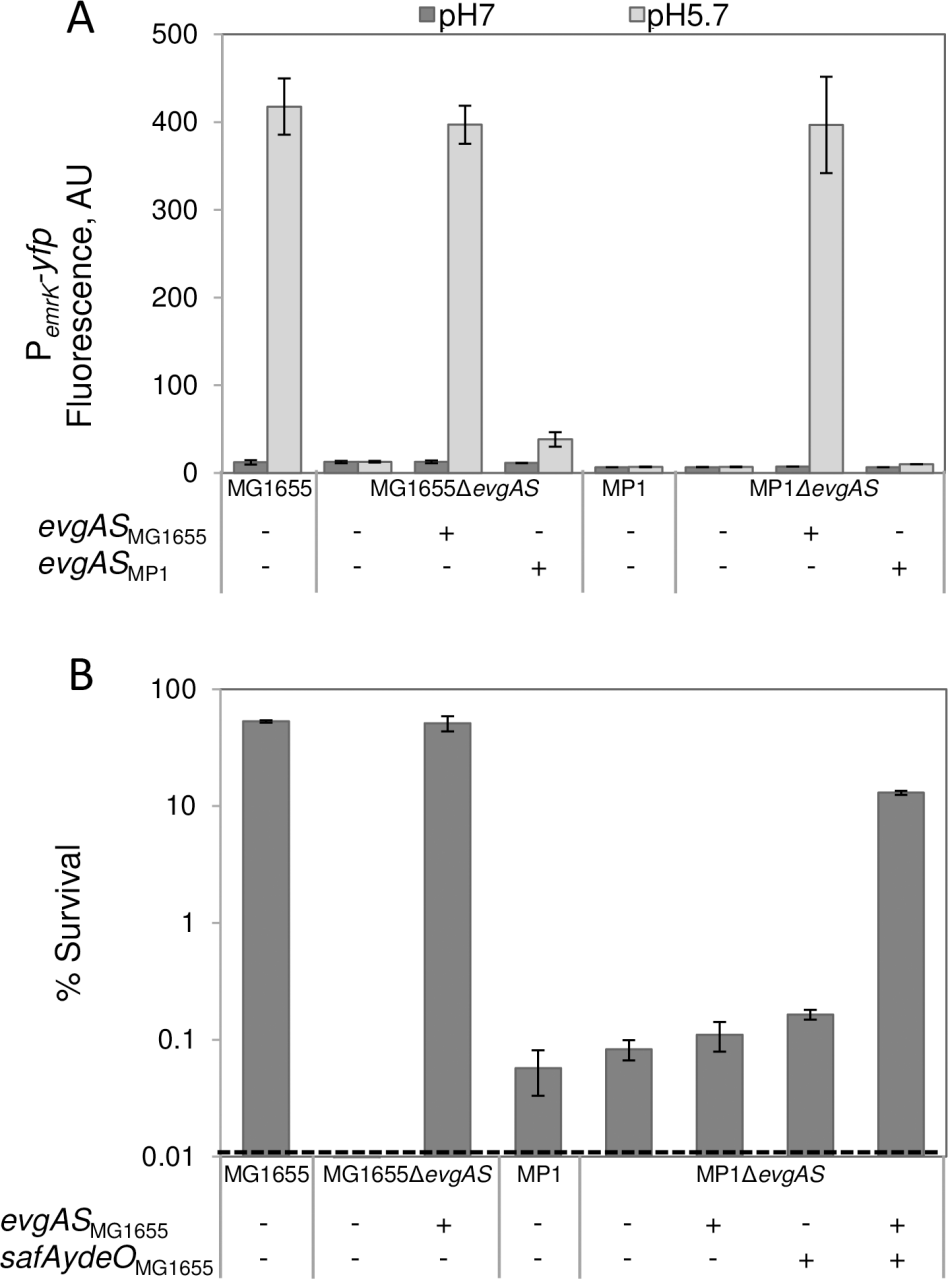
EvgAS_MG1655_ is sufficient to restore response to mild acidity in MP1. A: Activity of the fluorescent reporter P_*emrK*_*-yfp*. Strains with wild type *evgAS* (MMR182 and MP146) or Δ*evgAS* (MMR191 and MP162), and with the empty vector (pSMART) or plasmids p*evgAS*_MG1655_ (pMR78) or p*evgAS*_MP1_ (pMR84) were cultured in minimal medium at pH 5.7 or pH 7 to OD_600_ ~0.2, and fluorescence was quantified as described in Materials and methods. Values are the average fluorescence from two representative experiments and error bars represent the range. B: Resistance to acid challenge. Wild type or Δ*evgAS* strains derivatives of MG1655 (MMR182 and MMR191) and of MP1 (MP146 and MP162), and with the empty vector pSMART or plasmids p*evgAS*_MG1655_ (pMR78), p*safAydeO*_MG1655_ (pMR76), or p*evgAS*_MG1655_-*safAydeO*_MG1655_ (pMR128) were cultured in minimal medium at pH 5.7 to OD_600_~0.2, and shocked for an hour at pH 2.5 as described in Materials and methods. Percent survival values are the average of two representative experiments and error bars represent the range.

Based on the above results, we hypothesized that low pH might not function as an input signal for the EvgS/EvgA phosphorelay in MP1. We therefore measured pH induction of the *emrK* promoter, which is directly regulated by EvgA [4] (Fig 1A). We found that transcription was induced in MG1655, as expected, but not in MP1 (Fig 3C, S3 Fig). In contrast, the constitutively active EvgS1 mutant was able to induce the *emrK* reporter in MP1. The failure of low pH to activate EvgS in MP1 could be due to differences between the *evgAS* operon in MG1655 and in MP1, or due to an upstream factor required for acid-sensing that is missing or non-functional in MP1. To explore these possibilities, we compared the pH induction of P_*emrK*_*-yfp* in MP1 Δ*evgAS* and MG1655 Δ*evgAS* transformed with single-copy plasmids expressing the *evgAS* operon from one or the other strain (p*evgAS*_MG1655_ or p*evgAS*_MP1_). We found that p*evgAS*_MG1655_ restores pH induction of P_*emrK*_*-yfp* in both MG1655 Δ*evgAS* and MP1 Δ*evgAS* (Fig 4A). In contrast, p*evgAS*_MP1_ shows minimal pH induction in either strain, although p*evgAS*_MP1_ in MG1655 does show a small amount of induction, suggesting that there may be some factors outside of the *evgAevgS* operon that contribute to pH sensing. Overall, however, the above results indicate that the primary differences in pH response for the EvgS/EvgA systems in MG1655 and MP1 are due to differences in the sensor kinase and/or response regulator proteins themselves. Furthermore, since the EvgA amino acid sequences from MG1655 and MP1 are identical whereas the EvgS sequences differ at 39 residues, the different pH response in the two strains is likely due to polymorphisms in EvgS. This conclusion is further supported by the fact that a plasmid expressing a hybrid operon consisting of *evgA*_MP1_*evgS*_MG1655_ restores pH induction of P_*emrK*_*-yfp* in MG1655 Δ*evgAS* (S4 Fig).

The above results identify two properties of MP1 that potentially affect acid resistance: the chromosomal deletion containing *safAydeO* (Fig 1B) and differences in EvgS between MP1 and MG1655. To determine whether these factors account for the absence of inducible exponential phase acid resistance in MP1, we tested the survival of an MP1 derivative containing one or both of these loci from MG1655 following acid shock. Incorporation of both loci from MG1655 into MP1 (MP1 Δ*evgAS /* p*evgAS*_MG1655_ *-safAydeO*_MG1655_) rescued the inducible acid resistance phenotype by two orders of magnitude compared to MP1 with either plasmid p*safAydeO*_MG1655_ or plasmid p*evgAS*_MG1655_ (Fig 4B). These results indicate that in addition to the absence of the chromosomal segment containing *safAydeO*, sensitivity to acid shock in MP1 results from the inability of EvgS_MP1_ to respond to stimulation by mild acidity. We also note that the amino acid substitution F577S, which renders the EvgS_MG1655_ allele constitutively active (EvgS1), causes the same effect in EvgS_MP1_ (S5 Fig). This finding supports the hypothesis that although EvgS_MP1_ is expressed and functional, the protein cannot sense pH change.

The 39 residues in EvgS that differ between MG1655 and MP1 are distributed throughout the protein (S6 Fig). In an attempt to determine if a subset of these residues that are localized to a particular domain account for the pH insensitivity of EvgS_MP1_, we tested the activity of several EvgS hybrids containing swapped regions of EvgS_MP1_ and EvgS_MG1655_ (S4A Fig). These constructs were expressed from a single copy plasmid in a MG1655 Δ*evgS* strain, and activation of the EvgA-dependent reporter P_*emrK*_*-yfp* in response to acid stimulation was assessed. We found that all of the hybrids showed a strong pH-response (S4B Fig), indicating that pH insensitivity of EvgS_MP1_ cannot be ascribed to a single domain of EvgS_MP1_.

### Natural variation in pH sensing by the EvgS/EvgA phosphorelay among *E*. *coli*

**Fig 5:**
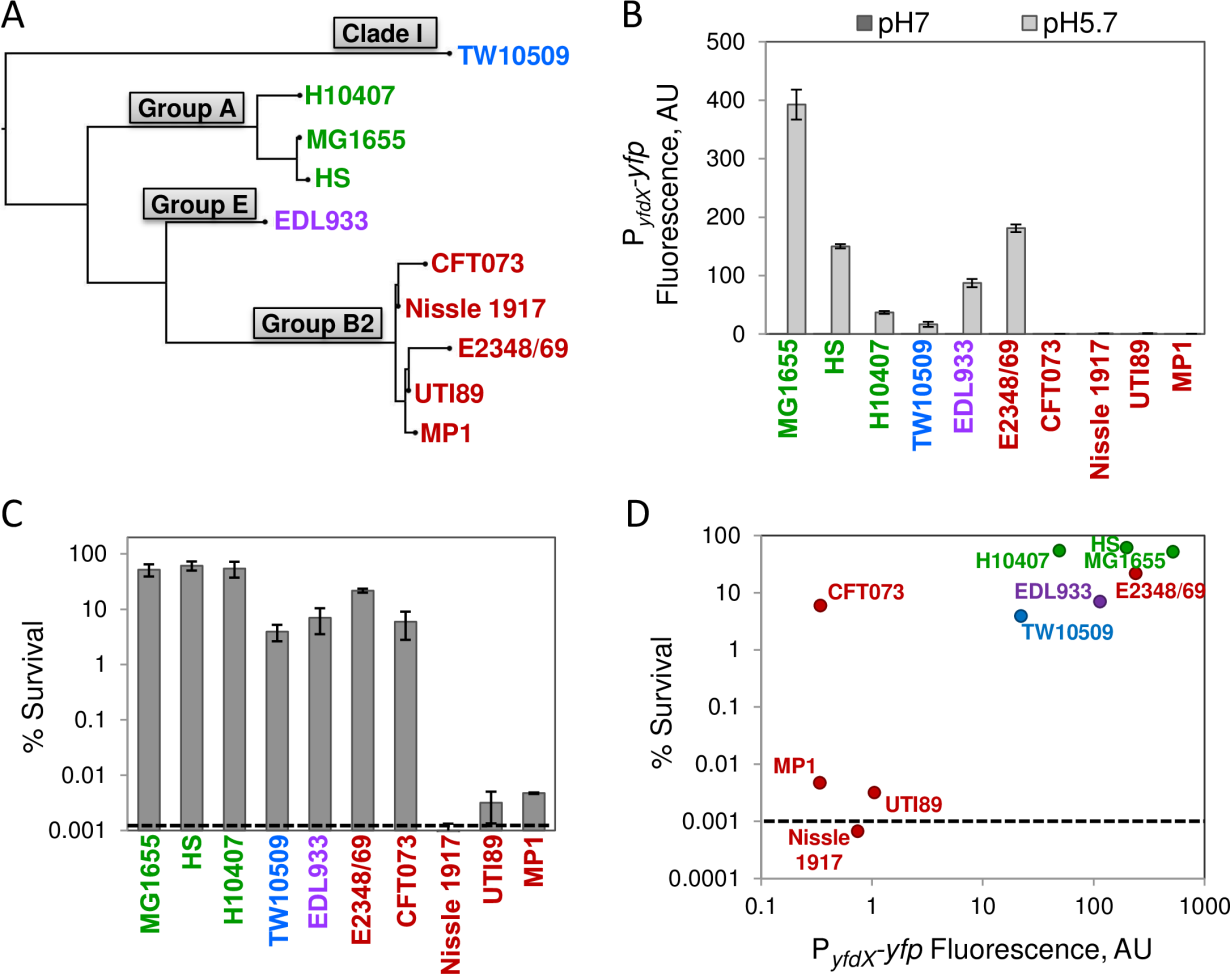
Analysis of EvgS and its sensitivity to mild acidity in selected *E*. *coli* isolates. A: Dendrogram based on EvgS sequence alignments was constructed as described in Materials and methods. Gray shaded boxes indicate phylogenetic groups. B: Activity of the EvgA fluorescent reporter P_*yfdX*_*-yfp*. All of the strains carry a single copy plasmid with the EvgA reporter P_*yfdX*_*-yfp*_MG1655_ (pMR86). The strains were cultured in minimal medium at pH 5.7 or pH 7 to OD_600_ ~0.2, and fluorescence was quantified as described in Materials and methods. Values are the average fluorescence from two experiments and error bars represent the range. All of the pH 7 data bars, and the pH 5.7 data bars for some strains, are too small to be visible in the figure. C: Resistance to acid challenge. Strains were cultured in minimal medium at pH 5.7 to OD_600_ ~0.2, and shocked for an hour in LB at pH 2.5 as described in Materials and methods. Percent survival values are the average of two experiments and error bars represent the range. D: Plot of fold induction of the fluorescent reporter P_*yfdX*_*-yfp* from panel B versus percent survival after exposure to pH 2.5, from panel C. The *E*. *coli* strains are described in detail in Table 1. Dashed lines represent the limit of detection of the assay.

Strains MG1655 and MP1 belong to different phylogenetic groups: A and B2, respectively [23]. We therefore wondered whether the properties noted above are unique to MP1 or are shared by other *E*. *coli* isolates. We considered eight representative strains (Table 1), which include commensals of group A and B2 (HS and Nissle respectively), intestinal pathogens (H10407, EDL933, and E2348/69), extra-intestinal pathogens (CFT073 and UTI89), and an “atypical” *E*. *coli* isolate classified in Clade I and of enterotoxigenic pathotype (TW10509). EvgS amino acid sequences from these strains have varying degrees of divergence from the MG1655 sequence (Table 1, S6 Fig), and a tree based on these sequences clusters according to each strain’s phylogenetic group (Fig 5A). A similar analysis that includes 285 EvgS sequences from fully sequenced *E*. *coli* genomes indicates that clustering according to the phylogenetic group is a general characteristic (S7 Fig).

**Table 1:** Characteristics of isolates used in this study.

Strain	Pathotype	Ecotype	Sample	Phylogeny	NCBI Taxid	% EvgS Identity
MG1655	Commensal	GIT	Human/Feces	Group A	511145	REF
HS	Commensal	GIT	Human/Feces	Group A	331112	99.92
H10407	ETEC	GIT	Human/Feces	Group A	316401	99.50
TW10509	ETEC	GIT	Human/Feces	*Escherichia* Clade I	656449	95.49
EDL933	EHEC	GIT	Food/Ground Beef	Group E	155864	97.83
E2348/69	EPEC	GIT	Human/Feces	Group B2	574521	96.41
Nissle 1917	Commensal	GIT	Human/Feces	Group B2	316435	96.74
CFT073	UPEC	UT	Human/Blood	Group B2	199310	96.57
UTI89	UPEC	UT	Human/Unknown	Group B2	364106	96.66
MP1	Commensal	GIT	Mouse/Feces	Group B2	1314835	96.74

Abbreviations: ETEC, Enterotoxigenic *E*. *coli*; EHEC, Enterohemorragic *E*. *coli*; EPEC, Enteropathogenic *E*. *coli*; UPEC, Uropathogenic *E*. *coli*; GIT, Gastrointestinal Tract; UT, Urinary Tract; REF, Reference.

Among the ten isolates, pairwise EvgS divergences are as large as 5.43%, and no two strains share 100% EvgS amino acid sequence identity (S8 Fig). This natural variation in EvgS is not a property of proteins encoded in neighboring genes: the EvgA amino acid sequence is identical across all ten strains, and YfdE, which is encoded by a gene just downstream of *evgS*, shows only a moderate level of divergence (S8 Fig). Additionally, the variation is not a general property of hybrid sensor kinases: two other hybrid kinases in *E*. *coli*, ArcB and BarA, are highly conserved among the ten isolates considered in this study (S8 Fig).

Based on the substantial sequence variation in EvgS, we hypothesized that the Evg systems in different *E*. *coli* strains would show varying responsiveness to acid pH that would be correlated with the degree of divergence from EvgS_MG1655_. To test this hypothesis, we assessed the pH induction of EvgS/EvgA in each strain using a single-copy plasmid containing a transcriptional fusion of *yfp* to the *yfdX* promoter (P_*yfdX*_*-yfp*), which is directly activated by phosphorylated EvgA [6]. We found that for group B2 strains, EvgS is not responsive to acid pH, with the exception of the EPEC strain E2348/69 (Fig 5B). The EvgS sequence from this isolate has the fewest substitutions (relative to MG1655) within the group B2 strains that we tested (Table 1). For strains outside the B2 clade, reporter expression varied from a 60-fold induction for HS, whose EvgS sequence is the closest to that of MG1655, to 8-fold induction in isolate TW10509.

We also compared the survival from acid shock for the various isolates following growth at pH 5.7 (Fig 5C). The pattern across the *E*. *coli* strains shows some correlation with EvgS sequence relatedness. Isolates in group A that were tested have comparably high resistance to acid challenge whereas the group B2 isolates Nissle, UTI89, and MP1 are quite sensitive. The association is imperfect however, as the two B2 isolates E2348/69 and CFT073 had significantly higher survival, albeit both were still more susceptible to acid shock than the group A strains (Fig 5C). Interestingly, CFT073 is very sensitive to slightly harsher acid shock (pH 2.25), as shown in S9A Fig. There is also a correlation between the extent of activation of the EvgS/EvgA system from growth at pH 5.7 (as assessed with a *yfdX* transcriptional reporter) and resistance to acid shock across the strains (Fig 5D). CFT073 is an exception to this trend for acid shock at pH 2.5, suggesting that this isolate has an exponential phase acid resistance pathway that does not require activation of the Evg system for this stress. However this strain clusters with the other closely related Group B2 isolates when shocked at pH 2.25 or lower pH (S9B Fig).

**Fig 6:**
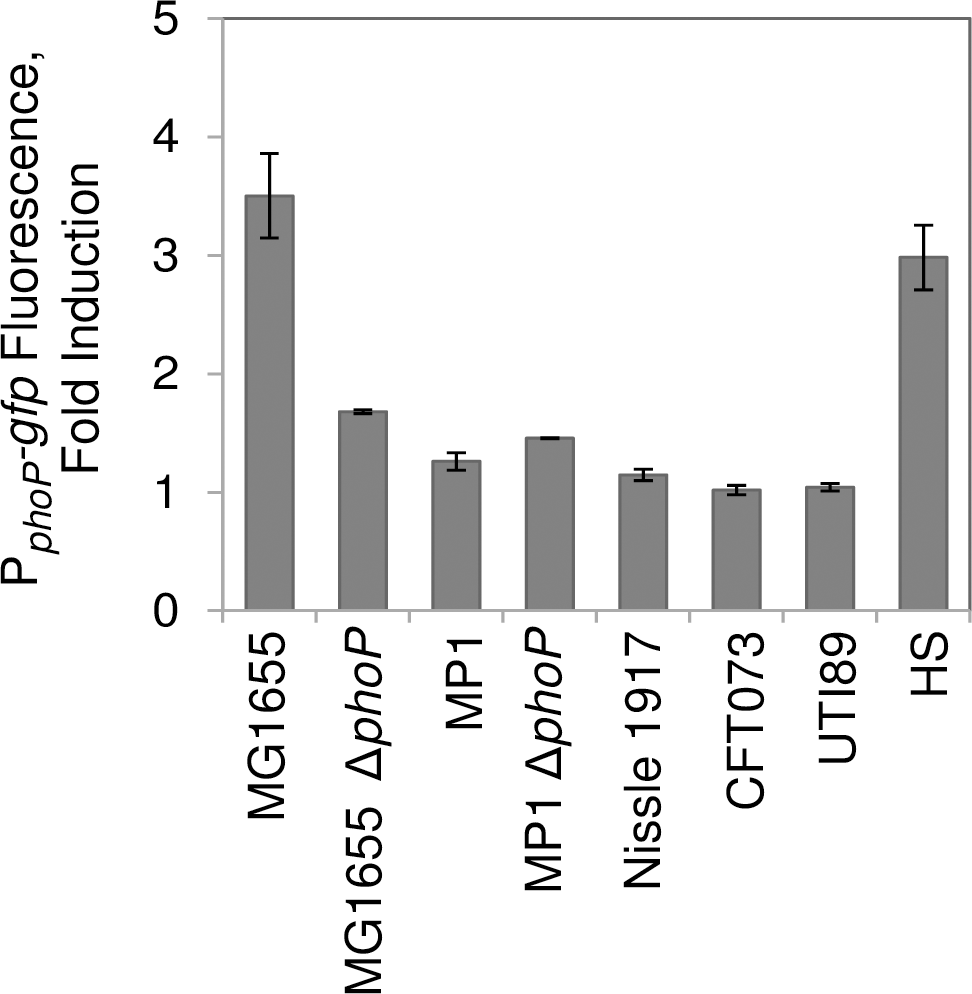
The PhoQ/PhoP system in group B2 isolates is not activated by mild acidity. Strains (in the same order as in the figure) MG1655, TIM136, MP1, MP200, Nissle 1917, CFT073, UTI89, HS containing a reporter plasmid with a PhoP-regulated promoter (P_*phoPQ*_*-gfp*) were cultured in minimal medium at pH 7 and pH 5.7, with 10 mM MgSO_4_, to OD_600_ ~0.2. Fluorescence of the reporter was measured as described in Materials and methods, and reported as fold induction from pH 7 to pH 5.7. Values are the average from two representative independent experiments. Error bars represent the range.

Above we showed that low pH fails to directly activate PhoQ/PhoP in MP1 (Fig 2A). Since we found that EvgS is not stimulated by mild acidity for three other group B2 isolates that we tested (Nissle 1917, CFT073, UTI89), we wished to determine if the PhoQ/PhoP system in these strains could be activated by low pH. To test this, we used a GFP reporter plasmid containing the PhoP-activated *phoPphoQ* promoter [25] and measured fluorescence of exponential phase cultures at pH 5.7 relative to pH 7. We found that like MP1, the three additional B2 strains that we tested showed no induction of the PhoP reporter (Fig 6), consistent with the conclusion that EvgS in these isolates is unresponsive to pH and that *E*. *coli* PhoQ is not directly stimulated by mild acidity (S1A Fig) [8].

### *evgAS* from MG1655 confers resistance to acid shock in acid-sensitive isolates

**Fig 7:**
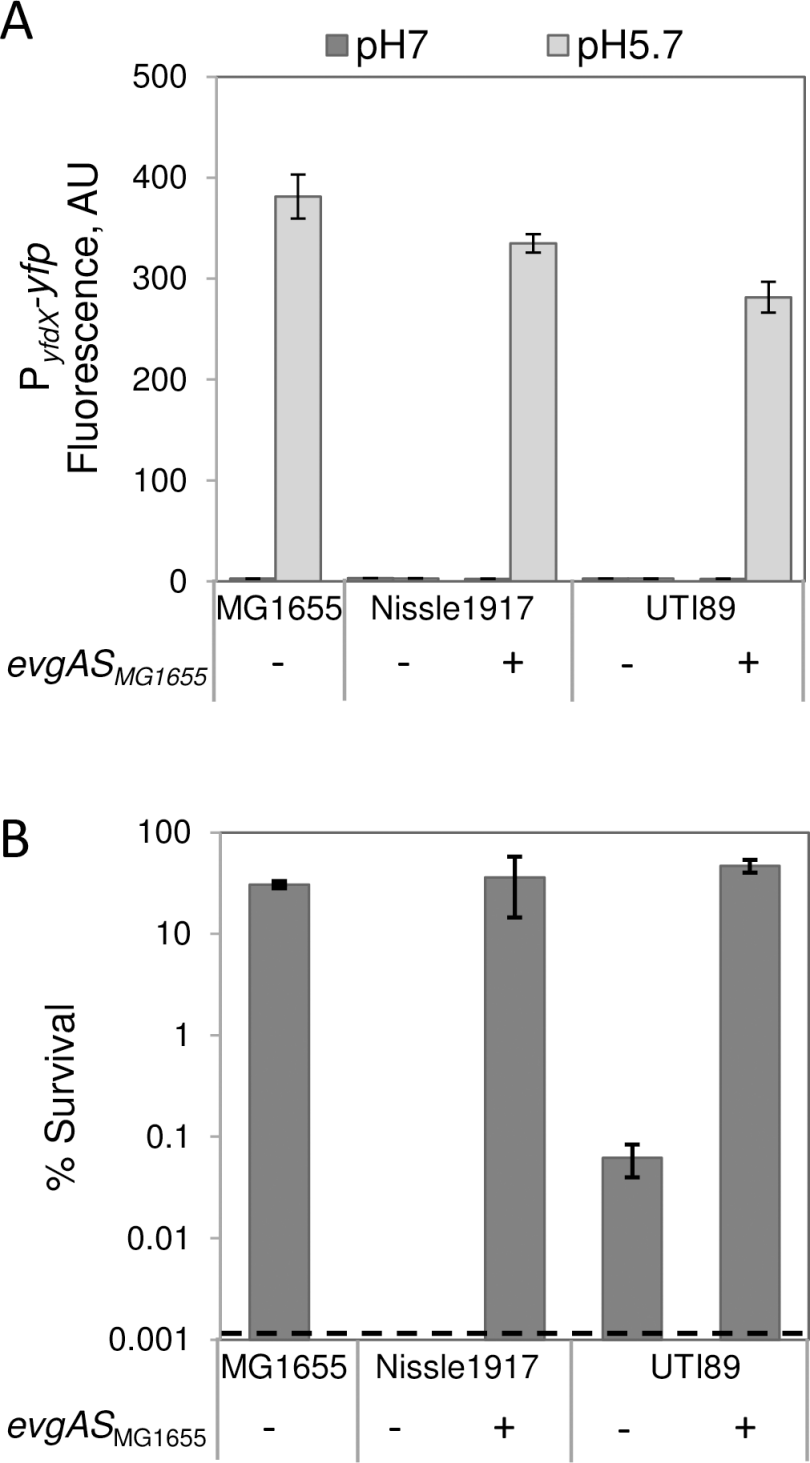
EvgAS_MG1655_ is sufficient to restore response to mild acidity in *E*. *coli* Nissle 1917 and *E*. *coli* UTI89. A: Activity of the fluorescent reporter P_*yfdX*_*-yfp*. Strains MMR178 (MG1655 P_*yfdX*_*-yfp)*, MMR227 (Nissle 1917 P_*yfdX*_*-yfp*), and MMR228 (UTI89 P_*yfdX*_*-yfp*), carrying the empty vector pSMART or p*evgAS*_MG1655_ (pMR78) were cultured in minimal medium at pH 5.7 or pH 7 to OD_600_~0.2, and fluorescence was quantified. Values are the average fluorescence from two representative experiments and error bars represent the range. B: Resistance to acid challenge. The same cultures as in (A) were shocked for an hour in LB at pH 2.5 as described in Materials and methods. Percent survival values are the average of two representative experiments and error bars represent the range. Dashed line represents the limit of detection of the assay.

The strains Nissle 1917 and UTI89 are similar to MP1 in their sensitivity to acid shock and inability to activate the Evg system in response to low pH (Fig 5). However, unlike MP1, these strains (as well as all of the other *E*. *coli* isolates that we tested) have an intact *safAydeO* locus. We therefore tested whether EvgS from MG1655 is sufficient to restore acid resistance in these strains by transforming each with the plasmid p*evgAS*_MG1655_. For both Nissle 1917/ p*evgAS*_MG1655_ and UTI89/ p*evgAS*_MG1655_, growth in mild acidity induced expression of the P_*yfdX*_*-yfp* reporter (Fig 7A). Likewise, in these strains EvgS_MG1655_ can activate the GadE-dependent reporter P_*hdeA*_*-yfp* (YdeO-dependent pathway) and the PhoQ/PhoP-dependent reporter P_*mgrB*_*-yfp* (SafA-dependent pathway) (S10 Fig), although the extent of activation is lower than that of MG1655. In addition, with the p*evgAS*_MG1655_ plasmid, both Nissle 1917 and UTI89 were as resistant to acid shock as MG1655 (Fig 7B). Thus, the pathway leading from mild pH induction to exponential phase acid resistance for strains UTI89 and Nissle 1917can be rescued with the *evgAevgS* operon from MG1655.

### Natural variation in *evgS* sequence is consistent with weak purifying selection

Since EvgA is identical across all of the *E*. *coli* strains used in this study, the above results suggest that the strain variability in pH response is due to differences in EvgS. We therefore analyzed the nucleotide sequence evolution of *evgS*. To test functional conservation across *evgS* orthologs, we calculated the d*N*/d*S* ratio, which is a measure of selection on protein-coding sequences [28, 29]. For any given gene, d*N*/d*S* is defined as the ratio of the average number of nucleotide substitutions per non-synonymous site (d*N*) to the average number of substitutions per synonymous site (d*S*). A d*N*/d*S* ratio that is less than one indicates purifying or stabilizing selection, d*N*/d*S* equal to one indicates neutral selection, and d*N*/d*S* greater than one indicates positive or adaptive selection.

To estimate d*N*/d*S*, *evgS* sequences were aligned using TranslatorX [30] and analyzed with the Synonymous Non-synonymous Analysis Program (SNAP v2.1.1) [31]. The d*N*/d*S* ratio calculated for the ten *E*. *coli* isolates is less than one (0.1), indicating that *evgS* is under purifying selection and, overall, is functionally conserved (Table 2, S4 Table).

**Table 2:** Sequence evolution of *evgS* based on d*N*/d*S* analysis.

	d*N*/d*S*^a^		
Gene	All 10 *E*. *coli* isolates	Group A	Group B2
*evgS*	0.10	0.32^b^, 0.44^c^	0.08^d^, 0.07^e^
*evgS* (1–537)	0.11	0.23	0.11
*evgS* (87–234)	0.11	0.45	ND^f^
*evgA*	0	ND^f^	0
*yfdE*	0.06	0	0.08
*barA*	0.01	0	0.01
*arcB*	0.01	ND^f^	0.02

^a^d*N*–Average number of substitutions per non-synonymous site; d*S*–Average number of substitutions per synonymous site; d*N*/d*S*–Ratio of rates of non-synonymous (d*N*) and synonymous (d*S*) substitutions, also known as Ka/Ks.

^b,d^values corresponding to the *E*. *coli* strains included in this study belonging to phylogenetic groups A and group B2, respectively

^c,e^values corresponding to all the *E*. *coli* strains from phylogenetic groups A and B2, respectively, derived from the MLST-based tree [23]

^f^ND–Not determined

To assess whether different clades experience varying selective pressures, we also calculated d*N*/d*S* for *evgS* within phylogenetic groups A and B2 separately. The group A isolates have a d*N*/d*S* ratio of 0.32, which is significantly higher than that of the group B2 isolates as well as the overall ratio for all ten organisms (~0.1). In addition, the Z-test for neutral selection (MEGA 4.0, [32]) gives a *p*-value = 0.2 for group A *evgS*. We therefore cannot reject the null hypothesis that d*N* = d*S*, suggesting that *evgS* from group A is under weaker purifying selection compared to that of B2. We observed a similar trend when we analyzed only the periplasmic region of *evgS* (1–537, 87–234) (Table 2, S4 Table).

We extended our d*N*/d*S* analysis to the two genes that flank *evgS* in the *E*. *coli* genome (*evgA* and *yfdE*) and also two other hybrid sensor kinases (*barA* and *arcB*) (Table 2, S4 Table). In contrast with *evgS*, these four genes appear to be under strict purifying selection, with similar values for d*N*/d*S* for all ten isolates together and for groups A and B2 separately.

To check if the d*N*/d*S* statistics estimated here are consistent across other *E*. *coli*, we calculated the d*N*/d*S* values for *evgS* as well as control genes for all complete *E*. *coli* genomes in the NCBI database. The resulting values closely match those obtained for the ten isolates (Tables 2, S4 and S5 Tables).

The EvgS/EvgA system in *E*. *coli* is homologous to the virulence-associated BvgS/BvgA system in *Bordetella pertussis* [33] and KvgS/KvgA system in *Klebsiella pneumoniae* [34]. We therefore tested whether these orthologs showed similar levels of variation as that of EvgS/EvgA. We repeated the d*N*/d*S* analyses for the *bvgAbvgS* and *kvgAkvgS* genes using the available complete sequences from *B*. *pertussis* and *K*. *pneumoniae*, respectively (S5 Table). For *bvgAbvgS*, the sequences from different isolates are highly invariant and showed very few non-synonymous or synonymous changes, if at all. This result is consistent with previous observations [35], and suggests that *bvgAbvgS* may either be recently acquired by *B*. *pertussis* and/or that the selective pressures to maintain the sequences of *bvgAbvgS* are strong, which is not surprising given the significant role of BvgS/BvgA system in virulence regulation.

In contrast with BvgS, the variation for *K*. *pneumoniae kvgS* is similar to that of *E*. *coli evgS* (S5 Table). However, whereas *E*. *coli evgA* shows very little variation relative to *evgS*, the variation of *K*. *pneumoniae kvgA* is comparable to that of *kvgS*, suggesting weaker selection for *kvgA* relative to *evgA*. In addition, the flanking gene *yfdX* in *K*. *pneumoniae* has a high level of variation as well, and the variations of all three genes *kvgA*, *kvgS*, *yfdX* are substantially higher than those of *K*. *pneumoniae arcB* and *barA*.

## Discussion

It is well established that the EvgS/EvgA system in the laboratory strain *E*. *coli* K-12 is stimulated by moderately acidic pH and that this response enables exponentially growing cells to resist severe acid shock [7, 8]. As shown here, this behavior extends to additional *E*. *coli* isolates—namely those in the same phylogenetic group as the K-12 strain, group A, as well as isolates from several additional groups (Fig 5). However, for other *E*. *coli* isolates, the Evg system is much less responsive, or not responsive at all, to acid pH. Furthermore, this behavior correlates with a decreased ability to survive extreme acid shock following growth in mild acidity (Fig 5). For the three strains with the largest difference in acid pH response relative to that of *E*. *coli* K-12—MP1, Nissle 1917, UTI89—we found that the primary differences in pH-sensing capacity are intrinsic to the EvgS/EvgA system itself, rather than arising from accessory proteins or other factors that vary between cells. For two of these strains (Nissle 1917 and UTI89), transformation with a K-12 EvgS/EvgA system led to acid resistance that was comparable to K-12. Thus, the suite of genes required to confer protection from acid stress is intact in these strains and is under control of the Evg system, despite the fact that the native system cannot be activated by mild acidity. In contrast, for the mouse commensal strain MP1, activation of EvgS/EvgA was not sufficient to provide protection from acid shock due to the absence of a chromosomal segment encoding the EvgA-regulated transcription factor YdeO and the connector protein SafA. Restoration of this chromosomal region from K-12, in combination with the K-12 EvgS/EvgA proteins, led to exponential phase acid stress resistance comparable to that of K-12. The absence of the *safAydeO* region in MP1 indicates that the Evg network has been significantly reduced, since YdeO is a key component of this network due to its control of GadE, which in turn regulates many downstream genes involved in acid resistance [6–8, 13, 26, 36]. In addition, since SafA functions as a connector between the Evg and PhoQ/PhoP systems, its absence disconnects the two pathways, abrogating activation of the PhoQ/PhoP system by signals that stimulate EvgS. However, we find that this branch of the Evg pathway does not play an important role in exponential phase acid resistance, at least for the acid stress assays that we employed.

Our results indicate that the diversity in pH response of the Evg system stems from natural variation in the EvgS sensor kinase. Furthermore, a d*N*/d*S* analysis indicates that *evgS* is under purifying selection, but that the selection is significantly weaker than that of the hybrid sensor kinase genes *arcB* and *barA*. This observation is consistent with the fact that the Evg system is broadly conserved in *E*. *coli* but that the ability of pH to function as an input signal is highly variable across isolates.

The pH-sensing mechanism of EvgS (for those EvgS variants that have this capacity) is not known. An analysis of EvgS mutants in MG1655 or related K-12 strains has implicated the periplasmic and cytoplasmic domains as playing a role in the pH response [9–11], although it remains to be established whether the sensing is direct or requires additional cellular components. Our own analysis of hybrid molecules in which domains of EvgS were substituted by analogous portions of the pH insensitive EvgS_MP1_ suggests that differences across multiple domains account for the absence of pH sensing in MP1. These observations are consistent with a recently proposed model of EvgS pH sensing in which pH modulates the strength of EvgS dimerization mediated by interactions in the periplasmic, transmembrane, and cytoplasmic domains [11]. In addition, from the EvgS amino acid sequence alignments (S6 Fig), we were unable to identify specific residues that likely account for the differences in pH-responsiveness of EvgS natural variants. For example, EvgS_E2348/69_, which is stimulated by low pH and is in group B2, differs from the four group B2 EvgS proteins that are not stimulated by pH at residues S382 (in the periplasmic domain), N859 (in the histidine kinase domain), and A1191 (at the C-terminus). However, strain E2348/69 is an exception since these residues are conserved across all of the nine other strains in this study. These observations indicate that multiple independent polymorphisms account for variability in pH sensing by EvgS.

The diversification of the Evg two-component system within *E*. *coli* suggests that pH is not the only input signal for the EvgS sensor kinase, and that the primary selective pressure for maintaining this signaling system may be associated with some other (unknown) stimulus. The fact that for some strains with a pH-nonresponsive EvgS the EvgA regulon still confers acid resistance further suggests the unknown input signal is strongly correlated with conditions of acid stress. The emergence of these EvgS variants may reflect selection for different dose-response behaviors with respect to other signals. However, since some isolates such as MP1 have evolved a network that disconnects acid resistance effectors from the Evg regulon (through loss of *safAydeO*), it seems likely that Evg input signals are not always correlated with acid stress.

The presence of the EvgS/EvgA system in the vast majority of *E*. *coli* isolates indicates there is a significant selective pressure to maintain this system, which makes the natural variation and genetic flexibility of EvgS all the more striking. This behavior stands in stark contrast with the common assumption that sensor kinases and response regulators of conserved two-component systems behave uniformly across a species (or even across closely related genera). Similar diversification may be present in other core signaling systems and poses a challenge for extrapolating from well-studied members of a bacterial species.

## Materials and methods

### Growth media and conditions

Liquid cultures were grown at 37°C in minimal A medium [37] supplemented with 0.2% glucose and 0.1% casamino acids, or in minimal A medium buffered at pH 5.7 with 100 mM 2-(N-morpholino)ethanesulfonic acid (MES, Sigma-Aldrich) and HCl. Minimal medium cultures of strain UTI89, which is auxotrophic for nicotinamide, and its derivatives were supplemented with 5 μg/ml nicotinamide. Bacterial cultures that were used to prepare electro-competent cells were grown in SOB with the appropriate antibiotic, when necessary. Cultures for preparing P1_vir_ lysates and for transductions were grown in LB (Miller) broth. LB-agar plates were used to grow cultures for CFU (colony forming unit) counts. To select for antibiotic resistance and to maintain plasmids, antibiotics were added to culture media to the following concentrations: ampicillin 50 μg/ml; for MG1655 and its derivatives—kanamycin 25 μg/ml, chloramphenicol 25 μg/ml or 12.5 μg/ml for single copy plasmids (pSMART derivatives); for all other strains—chloramphenicol 12 μg/ml or 6 μg/ml for single copy plasmids (pSMART derivatives); kanamycin 50 μg/ml for Nissle 1917 and its derivatives, 35 μg/ml for all other strains.

### Strains and plasmids

Strains and Plasmids used in this study are described in S1 and S2 Tables, respectively. Primers used in this study are listed in S3 Table. Transformations of plasmids and linear DNA for chromosomal integration were performed by electroporation. Antibiotic cassettes flanked by FRT sites were removed, when necessary, with the plasmid pCP20 as described in [38]. Transductions were conducted with phage P1_vir_ [37]. For details on strain and plasmid constructions, see S1 Methods.

### Fluorescence quantification

Fluorescence was quantified by microscopy. Cultures were inoculated from single colonies on LB Agar plates and grown in minimal medium aerobically at 37°C overnight to saturation, then diluted 1:1000 into fresh medium that was at pH 7 or pH 5.7 (as indicated). To test PhoQ/PhoP activation via stimulation of EvgA and the connector SafA and to avoid stimulation by low magnesium, overnight cultures were diluted in minimal medium with 10 mM MgSO_4_. Conversely, to activate PhoQ/PhoP with low magnesium, overnight cultures were diluted in minimal medium at pH 7 and containing 1 μM MgSO_4_. Cultures were grown at 37°C to an optical density at 600 nm (OD_600_) of 0.2–0.3, then rapidly cooled in an ice-water slurry and kept on ice for at least 1 hour. Fluorescence microscopy and data analysis were performed as previously described [39]. For each data set, the fluorescence of at least one hundred cells was recorded. The mean fluorescence from each data set was background subtracted, and the average between replicas was calculated.

### Acid resistance assay

Cultures were grown as described above for fluorescence microscopy to OD_600_ of 0.2. Cultures were concentrated to one sixth of the volume by centrifugation. Because of the low density of the cultures, this step was necessary to lower the limit of detection of viable cells following acid challenge. Fifty microliters of the concentrated cultures (approximately 1-5x10^7^ cells) were transferred to 1 ml sterile phosphate-buffered saline (PBS 137mM NaCl, 2.7mM KCl, 10mM Na_2_HPO_4_, 2mM KH_2_PO_4_), pH 7.4, and 50 μl were transferred to 1 ml of LB broth, pH 2.5 (acidified with HCl), that was pre-warmed to 37°C. The bacterial suspensions in acidified LB broth were incubated at 37°C for one hour, or the indicated times. Both LB and PBS cell suspensions were serially diluted in PBS, and aliquots were immediately plated in triplicate. After incubation overnight, CFUs were counted. The percentage survival was calculated as the number of CFUs/ml of acid shocked cultures divided by the number of CFUs/ml of the cultures diluted in PBS. For S9A Fig, the experiments were conducted in the same way, except the cultures were exposed to LB at pH 2.25, 2.0, or 1.75 as indicated.

### Sequence alignments and phylogenetic analysis

Full-length EvgS, EvgA, YfdE, BarA, and ArcA sequences were extracted from complete *E*. *coli* genomes in NCBI using tblastn (http://blast.ncbi.nlm.nih.gov) using the corresponding sequences from *E*. *coli* K-12. Sequence alignments of S6 and S8 Figs were obtained with Clustal Omega, [40, 41] on the EMBL-EBI server. EvgS sequences in Fig 5A and S7 Fig were aligned and neighbor-joining trees were constructed using Muscle [42] on the EMBL-EBI server with the default parameters and displayed using Figtree (http://tree.bio.ed.ac.uk/software/figtree/) with midpoint rooting. For S7 Fig, the phylogenetic groups for the corresponding *E*. *coli* isolates from which these EvgS sequences were derived were determined as described in [43].

### d*N*/d*S* analysis to assess nucleotide divergence

Nucleotide sequences for the genes of interest were obtained from the NCBI database. Multiple sequence alignments were generated in a codon-delimited format on the TranslatorX server [30] using MUSCLE alignment software [42]. To obtain gene-specific d*N*/d*S* estimates, we utilized the Synonymous Non-synonymous Analysis Program (SNAP v2.1.1) implementing Nei and Gojobori’s method [28] and its statistic output tool [31] available on the HIV sequence database website (www.hiv.lanl.gov, Korber, 2000). The codon-aligned nucleotide sequence alignments (.aln or fasta file) were provided as input into the SNAP tool to compute d*N*/d*S* ratios. Alignments were imported into MEGA software v4.0 [32] to perform a Z-test of neutral selection, for a null hypothesis (d*N* = d*S*) using modified Nei-Gojobori (Jukes-Cantor) method.

## Supporting information

S1 Methods Construction of strains and plasmids used in this study. (PDF)

S1 Table List of bacterial strains used in this study. (PDF)

S2 Table List of plasmids used in this study. (PDF)

S3 Table Primers used in this study. (PDF)

S4 Table Sequence evolution of *evgS* based on d*N*/d*S* analysis. (PDF)

S5 Table d*N*/d*S* analysis for *evgS* (*Escherichia coli*), *bvgS* (*Bordetella pertussis*), *kvgS* (*Klebsiella pneumoniae*) and related genes based on currently available complete genomes at NCBI. (PDF)

S1 Fig Activation of the PhoQ/PhoP system from different stimuli. A: The PhoQ/PhoP system is not activated by mild acidity over a range of pH values. Transcriptional reporter derivatives of MG1655 (TIM92), MG1655 Δ*safA* (SAM74), and MP1 (MP131) were cultured to OD_600_ ~0.2 in minimal medium containing 10 mM MgSO_4_ and buffered with 100 mM MES, at pH 5.1, 5.3, 5.5, 5.7, 5.9, 6.1, 6.3, and 7. Fluorescence was determined as described in Materials and methods. Fluorescence values are the average from two independent experiments. Error bars represent the range. B: *mgrB* is activated in MP1 by low Mg^++^ similarly to *E*. *coli* K-12. Strains TIM63 (MG1655 P_*mgrB*_*-yfp)* and MP131 (MP1 P_*mgrB*_*-yfp*) were cultured to OD_600_ ~0.2 in minimal medium at pH 7 with either 10 mM or 1 μm MgSO_4_. Fluorescence was determined as described in Materials and methods. Fluorescence values are the average from two independent experiments. Error bars represent the range.
(PDF)

S2 Fig Resistance to acid shock. A: resistance of strains MG1655 and MP1 in different growth stages. Strains were cultured in minimal medium at pH 7 to stationary phase (16 hours) and at pH 5.7 to exponential phase (OD_600_ ~0.2). Cultures were shocked for an hour at pH 2.5 as described in Materials and methods. Values are the average percent survival from two representative experiments and error bars represent the range. B: Resistance of MP1*, a strain derived from MP1 by transducing from MG1655 a region encompassing the 13 kb segment that is absent in MP1. Strains MG1655, MP1 and MP1* (MP144) were cultured at pH 5.7 to exponential phase (OD_600_ ~0.2), and acid resistance was assayed as in A. Values are the average percent survival from two representative experiments and error bars represent the range. C: Strains MG1655, MMR241 (MG1655 Δ*evgAS*), TIM96 (MG1655 Δ*phoQP*), and SAM74 (MG1655 Δ*safA*) were cultured and assayed for acid resistance as in B, except samples were withdrawn at the indicated times. Values are the average percent survival from two representative experiments and error bars represent the range.
(PDF)

S3 Fig EvgS_MP1_ is not responsive to mild acidity in the pH range effective for EvgS_MG1655_. Strains MMR182 (MG1655 P_*emrK*_*-yfp)* and MP146 (MP1 P_*emrK*_*-yfp*) were cultured in minimal medium at pH 7 or in minimal medium buffered with 100 mM MES at pH 5.1, 5.3, 5.5, 5.7, 5.9, 6.1, 6.3, and 7. Cultures were harvested at OD_600_~0.2, and fluorescence of the reporter was measured as described in Materials and methods. Fluorescence values are the average from two representative independent experiments. Error bars represent the range.
(PDF)

S4 Fig EvgS domain swap between MG1655 and MP1. A: Diagram illustrating the plasmids and hybrid EvgS proteins utilized in this assay. Predicted transmembrane domains (TM), and residues they span, are indicated. (*) indicates a transmembrane domain that is predicted in some databases (www.uniprot.org) but is inconsistent with a homology model based on the ortholog BvgS [11]. Residues delimiting the swapped EvgS regions are numbered at the bottom and indicated by the dotted lines. B: Activity of the EvgA-dependent reporter P_*emrK*_*-yfp*. The hybrid names correspond to those indicated in panel A. Strains with either wild type *evgAS* (MMR182) carrying the empty vector (pSMART), or Δ*evgAS* (MMR191) with the empty vector, or one of the plasmids pMR78, pMR117, pMR84, pMR80, pMR92, pMR82, pMR83 (in this order in the figure) were cultured in minimal medium at pH 7 and pH 5.7 to OD_600_~0.2. Fluorescence was quantified by microscopy as described in Materials and methods. Values are the average fluorescence from two independent experiments and error bars represent the range.
(PDF)

S5 Fig The single residue change that makes EvgS_MG1655_ constitutively active has a similar effect on EvgS_MP1_. Derivatives of MG1655 and MP1 with the P_*emrK*_*-yfp* reporter and with WT *evgS* (MMR182 and MP146) or with the the reporter and the F577S amino acid substitution associated with the *evgS1* allele (MMR183 and MP145) were cultured in minimal medium at pH 5.7 (induced wild-type strains) and pH 7 (F577SEvgS) strains and non-induced wild-type strains) to OD_600_~0.2 and fluorescence of the reporter was measured as described in Materials and methods. Fluorescence values are the average from two representative independent experiments. Error bars represent the range.
(PDF)

S6 Fig Alignment of EvgS from ten *E. coli* isolates. EvgS protein sequences from the indicated ten *E*. *coli* isolates were aligned as described in Materials and methods. (*) indicates a predicted transmembrane domain (www.uniProt.org) that is inconsistent with a homology model based on the ortholog BvgS [11]. The predicted PAS domain is shaded gray, and the green highlights indicate residues identified in previous studies as being involved in EvgS activity [4, 9–11].
(PDF)

S7 Fig EvgS phylogeny in *E. coli* isolates. Dendrogram based on EvgS sequences from 285 *E*. *coli* genomes. The colors correspond to phylogenetic groups: red–group A, orange–group C, green–group B1, light blue–group D, dark blue–group E, purple–group B2. The phylogenetic groups are also appended to the genome identifiers in the figure. Full-length EvgS sequences were extracted from complete *E*. *coli* genomes in NCBI using tblastn (http://blast.ncbi.nlm.nih.gov) with the *E*. *coli* K-12 EvgS sequence, and the large number of redundant hits from the genomes of *E*. *coli* K-12 derivatives were removed, resulting in 285 EvgS sequences. The tree construction is described in Materials and methods.
(PDF)

S8 Fig Protein divergence matrices of ten *E*. *coli* isolates. The indicated protein sequences from ten *E*. *coli* isolates were aligned as described in Materials and methods. Matrix entries indicate the percentage of amino acids that differ for the corresponding pairs of strains. In addition to EvgS and EvgA, matrices are shown for YfdE, a protein encoded by a gene adjacent to *evgS*, and for two other hybrid histidine kinases, BarA and ArcB. All five matrices use the indicated color range scale.
(PDF)

S9 Fig Sensitivity of selected *E*. *coli* isolates to acid challenge in a range of pH values. A: indicated strains were cultured in minimal medium at pH 5.7 to OD_600_ ~0.2, and shocked for an hour in LB at pH 2.25 or pH 2.0, or pH 1.75 as described in Materials and methods. Percent survival values are the average of two representative experiments and error bars represent the range. As a reference, the figure includes the values of challenge at pH 2.5 from Fig 5C. B: Correlations between fold induction of the fluorescent reporter P_*yfdX*_*-yfp* from Fig 5B versus percent survival after exposure to pH 2.25, pH 2.0, and pH 1.75 from panel A. The *E*. *coli* strains are described in detail in Table 1. Dashed lines represent the limit of detection of the assay.
(PDF)

S10 Fig EvgAS_MG1655_ rescue of the SafA and YdeO pathways in *E*. *coli* Nissle 1917 and *E*. *coli* UTI89. Derivatives of MG1655, Nissle1917 and UTI89 with the P_*hdeA*_*-yfp* reporter (Panel A, MMR175, MMR254 and MMR255) or with the P_*mgrB*_*-yfp* reporter (Panel B, TIM95, MMR272 and MMR273), and carrying the empty vector pSMART or its derivative p*evgAS*_*MG1655*_ (pMR78), were cultured in minimal medium at pH 7 and pH 5.7 to OD_600_~0.2. Fluorescence of the reporter was measured as described in Materials and methods. Fluorescence values are the average from two representative independent experiments. Error bars represent the range.
(PDF)
