# Supplementary material for: Natural variation of a sensor kinase controlling a conserved stress response pathway in Escherichia coli
Source: PLoS Genet. 2017 Nov 15;13(11):e1007101. doi: 10.1371/journal.pgen.1007101 (PMC5706723; doi:10.1371/journal.pgen.1007101)
Supplement: S1 Methods — (PDF) [file pgen.1007101.s001.pdf]

Supplemental methods

Strains and Plasmids Construction

The chromosomal P_{hdeA} –*yfp,* P_{emrK} –*yfp,* P_{yfdX} –*yfp* transcriptional fusion fluorescent reporters were constructed by recombineering (Datsenko and Wanner 2000). Regions of approximately 500 bp upstream of the transcription start sites of *emrK*$_{MG1655}$, *emrK*$_{MP1}$ (Eguchi, Oshima et al. 2003), *hdeA*$_{MG1655}$, *hdeA*$_{MP1}$, and *yfdX*$_{MG1655}$ (Itou, Eguchi et al. 2009) were amplified by PCR. Primers were designed with restriction sites for cloning the amplified DNA in the corresponding *Bam*HI-*Kpn*I restriction sites of pEB45 (Batchelor and Goulian 2006), upstream of the ribosome binding site and *yfp* coding sequence. The plasmids thus obtained, pMR69 (with $P_{emrK\text{-MG1655}}$), pMR70 (with $P_{emrK\text{-MP1}}$), pMR71 (with $P_{hdeA\text{-MG1655}}$), pMR72 (with $P_{hdeA\text{-MP1}}$) and pMR73 (with $P_{yfdX\text{-MG1655}}$), were used as templates to generate a PCR product for integration in the *lac* operon. These PCR products had flanking regions of homology to sequence upstream of the *lacI* promoter, and in the *lacA* gene, and included the promoter-*yfp* transcriptional fusions and the kanamycin cassette flanked by FRT sites. The integrated constructs were verified by sequencing.

The allele *evgS1* has a single nucleotide substitution (nucleotide 1730) resulting in the amino acid change F577S in the linker region of the protein (Kato, Ohnishi et al. 2000). We introduced this change in *evgS* of MG1655 by two-step recombineering using a scarless mutagenesis protocol (Blank, Hensel et al. 2011) essentially as described. Briefly, a chloramphenicol (Cam) cassette and adjacent I-*Sce*I restriction site were amplified by PCR from template pWRG100 (Blank, Hensel et al. 2011), and primers with a 5' region of homology to *evgS*. The PCR product was integrated in the chromosome of MG1655 as described (Datsenko and Wanner 2000) with

selection for chloramphenicol resistance. In the second recombination step, an overlap extension PCR product containing *evgS* sequence flanking the I-*Sce*I-Cam cassette, and containing the T to C change at position 1730, replaced the I-*Sce*I-Cam cassette. Clones that lost the cassette were selected by expressing the anhydrotetracycline-inducible I-*Sce*I restriction enzyme from pWRG99, with subsequent loss of the non-recombinant cells. The resulting strain MMR165 was chloramphenicol sensitive and was sequenced to confirm the presence of the desired mutation.

To move the allele *evgS1* to MP1 strains, we first introduced into MMR165 a kanamycin resistance gene insertion in *torI*, which lies on the chromosome approximately eight thousand base pairs upstream of *evgS*. The genomic region containing Δ*torI*:: *FRT-kan-FRT* was PCR amplified from JW5387 (Baba, Ara et al. 2006) and integrated in MMR165 (Datsenko and Wanner 2000) to generate strain MMR166. The kanamycin cassette and linked *evgS1* were moved by transductions to all MP1 derivatives used in this study. After selection for the antibiotic resistance, the strains thus obtained were sequenced to verify the presence of MG1655 *evgS* and the *evgS1* mutation. We also verified that the Δ*torI::Kan*, did not affect EvgS activity, output, or resistance to acid shock in the conditions used in this work, as shown the figure below.

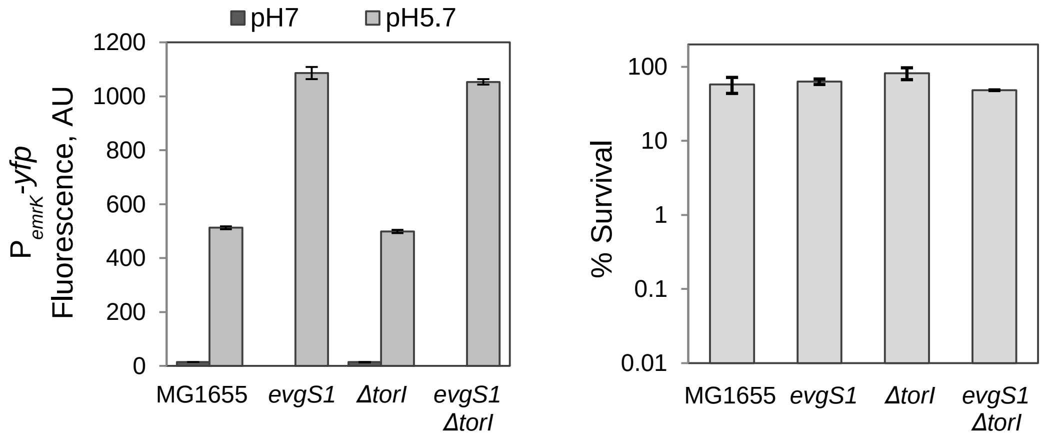

The *torI* deletion used as linked marker for transduction does not affect EvgS activity or acid resistance in MG1655. A: Δ*torI::kan* was used as a linked marker to transduce *evgS1*. Strains were cultured in minimal medium at pH 5.7 and pH 7 (MMR182 and MMR237) or at pH 7 (MMR183 and MMR236) to OD_{600}~0.2. Left panel, activity of the *evgA*-dependent promoter as fluorescence of the P_{emrK}-*yfp* reporter. Values are the average fluorescence from two representative experiments and error bars represent the range. Right panel, resistance to acid challenge in LB at pH 2.5 for one hour. Percent survival values are the average of two representative experiments and error bars represent the range.

The mutation F577S in $EvgS_{MP1}$ was constructed by recombineering (Datsenko and Wanner 2000). Briefly, we generated an overlap extension PCR product of $evgS_{MP1}$ containing the T to C change at position 1730, followed by a Kanamycin cassette at its 3'end. The PCR product was integrated in the chromosome of MP1 as described (Datsenko and Wanner 2000) with selection for kanamycin resistance. The Kanamycin cassette was inserted in the intergenic region between *evgS* and the neighboring gene *yfdE.* The presence of the codon change corresponding to the F577S mutation was verified by sequencing.

To construct strain MP1* we moved into MP1 the missing region of DNA containing *safAydeO* from MG1655 (Fig 1B) by P1 transduction. We used a strain with a kanamycin cassette inserted in place of *yneL*, JW5244 (Baba, Ara et al. 2006). This ORF encodes a peptide of unknown function. We verified that the deletion of *yneL* did not affect acid shock resistance in MG1655 for the conditions used in this work (not shown). The resulting MP1* strain (MP144) was verified by PCR to have acquired from MG1655 the missing chromosomal segment.

Deletions of *evgAS* and *safA* were created essentially as described (Datsenko and Wanner 2000). For *evgAS*, the *FRT-kan-FRT* cassette replaced the entire region from the second codon of *evgA* to the 7th to the last codon of *evgS*. Deletion of *ydeO* was obtained by P1 transduction from strain JW1494 of the KEIO collection (Baba, Ara et al. 2006)

To construct plasmid pMR76 with the *safAydeO* operon (*safAydeO* $_{MG1655}$), a region of genomic DNA spanning from 320 bp upstream of the *safA* start codon to 280 bp downstream of the *ydeO* stop codon was PCR amplified from MG1655. This region included the *safA* promoter and the regulatory region *evgA* consensus sequence (Masuda and Church 2003). The primers contained restriction sites so that the PCR product could be cloned into the *Bam*HI and *Hind*III sites of the single copy plasmid pSMART (Lucigen Corporation). The resulting plasmid pMR76 (p*safAydeO*$_{MG1655}$) was sequenced and the entire *safAydeO* locus sequence was verified to be correct.

To construct plasmids pMR78 and pMR84, which carry the *evgAS* operon from MG1655 or MP1 respectively (p*evgAS*$_{MG1655}$ and p*evgAS*$_{MP1}$), a region of genomic DNA spanning from 300 bp upstream of the *evgA* start codon to 18 bp downstream of the *evgS* stop codon was PCR amplified from MG1655 and from MP1. This region included the *evgA* promoter and the regulatory region *evgA* consensus sequence (Masuda and Church 2003). The primers contained restriction sites so that the PCR products could be cloned into the *Eco*RI and *Bam*HI sites of the single copy plasmid pSMART (Lucigen Corporation). The resulting plasmids pMR78 (p*evgAS*$_{MG1655}$) and pMR84 (p*evgAS*$_{MP1}$) were sequenced and the entire *evgAS* locus sequence was verified to be correct.

To construct plasmid pMR128 with the *safAydeO* operon and the *evgAS* operon (p*safAydeO* $_{\text{MG1655}}$-*evgAS*$_{\text{MG1655}}$), a PCR product containing *safAydeO* as in pMR76 was cloned downstream of *evgAS* into *Bam*HI and *Hind*III restriction sites of pMR78.

Plasmids pMR80, pMR82, pMR83, and pMR92 are derivatives of pMR78 with regions of *evgS* exchanged with the corresponding regions from MP1. pMR117 is a derivative of pMR84 with the entire *evgS* exchanged with the corresponding regions from MG1655. Hybrid DNA molecules were generated by overlap extension PCR.

Reporter plasmid pMR86 was constructed by PCR amplification of $P_{yfdX\text{-MG1655}}$-*yfp* from pMR73 , followed by cloning of the PCR products into *Eco*RI and *Bam*HI restriction sites of pSMART. The promoter and *yfp* sequences were verified to be correct.

References

Baba, T., T. Ara, et al. (2006). "Construction of Escherichia coli K-12 in-frame, single-gene knockout mutants: the Keio collection." Mol Syst Biol **2**: 2006 0008.

Batchelor, E. and M. Goulian (2006). "Imaging OmpR localization in Escherichia coli." Mol Microbiol **59**(6): 1767-1778.

Blank, K., M. Hensel, et al. (2011). "Rapid and highly efficient method for scarless mutagenesis within the Salmonella enterica chromosome." PLoS One **6**(1): e15763.

Datsenko, K. A. and B. L. Wanner (2000). "One-step inactivation of chromosomal genes in Escherichia coli K-12 using PCR products." Proc Natl Acad Sci U S A **97**(12): 6640-6645.

Eguchi, Y., T. Oshima, et al. (2003). "Transcriptional regulation of drug efflux genes by EvgAS, a two-component system in Escherichia coli." Microbiology **149**(Pt 10): 2819-2828.

Itou, J., Y. Eguchi, et al. (2009). "Molecular mechanism of transcriptional cascade initiated by the EvgS/EvgA system in Escherichia coli K-12." Biosci Biotechnol Biochem **73**(4): 870-878.

Kato, A., H. Ohnishi, et al. (2000). "Transcription of emrKY is regulated by the EvgA-EvgS two-component system in Escherichia coli K-12." Biosci Biotechnol Biochem **64**(6): 1203-1209.

Masuda, N. and G. M. Church (2003). "Regulatory network of acid resistance genes in Escherichia coli." Mol Microbiol **48**(3): 699-712.
