# Supplementary material for: Natural variation of a sensor kinase controlling a conserved stress response pathway in Escherichia coli
Source: PLoS Genet. 2017 Nov 15;13(11):e1007101. doi: 10.1371/journal.pgen.1007101 (PMC5706723; doi:10.1371/journal.pgen.1007101)
Supplement: S1 Table — (PDF) [file pgen.1007101.s002.pdf]

S1 Table. List of bacterial strains used in this study.

Strain	Relevant Genotype	Reference or Source
MG1655	F^- λ^- ilvG- rfb-50 rph-1	*E. coli* Genetic Stock Center, CGSC no. 7740
HS		(Rasko, Rosovitz et al. 2008)
Nissle 1917		(Grozdanov, Raasch et al. 2004)
H10407		(Crossman, Chaudhuri et al. 2010)
TW10509		(Walk, Alm et al. 2009; Luo, Walk et al. 2011)
TUV93-0	EDL933 *slt*1- *slt*2-	(Campellone, Giese et al. 2002)
E2348/69		Public Health England, (Iguchi, Thomson et al. 2009)
CFT073		(Mobley, Green et al. 1990)
UTI89		(Mulvey, Schilling et al. 2001)
MP1		(Lasaro, Liu et al. 2014)
SAM74	MG1655 Δ*safA*::(FRT-kan-FRT) att_{λ}::(P_{mgrB}-*yfp*) att_{HK022}(P_{tetA}-*cfp*)	This study
TIM63	MG1655 att_{λ}::(P_{mgrB}-*yfp*)-cam	(Miyashiro and Goulian 2007)
TIM92	MG1655 att_{λ}::(P_{mgrB}-*yfp*) att_{HK022}(P_{tetA}-*cfp*)	(Miyashiro and Goulian 2007)
TIM95	MG1655 att_{λ}::(P_{mgrB}-*yfp*)-kan	Goulian lab, unpublished
TIM96	MG1655 att_{λ}::(P_{mgrB}-*yfp*) att_{HK022}(P_{tetA}-*cfp*) Δ*phoQP*::FRT	Goulian lab, unpublished
TIM136	MG1655 Δ*phoP*::FRT	Goulian lab, unpublished

MMR165	MG1655 *evgS1* (F577SEvgS$_{MG1655}$)	This study
MMR166	MG1655 *evgS1* Δ*torI*::(*FRT-kan-FRT*)	This study
MMR170	MG1655 *evgS1* *att*$_{\lambda}$::(P$_{mgrB}$*-yfp*)	This study
MMR173	MG1655 Δ(*lacI lacZYA*)::(P$_{emrK}$*-yfp)-FRT-kan-FRT*	This study
MMR175	MG1655 Δ(*lacI lacZYA*)::(P$_{hdeA}$*-yfp)-FRT-kan-FRT*	This study
MMR178	MG1655 Δ(*lacI lacZYA*)::(P$_{yfdX}$*-yfp)-FRT-kan-FRT*	This study
MMR179	MG1655 *evgS1* Δ(*lacI lacZYA*)::(P$_{emrK}$*-yfp)-FRT-kan-FRT*	This study
MMR180	MG1655 *evgS1* Δ(*lacI lacZYA*)::(P$_{hdeA}$*-yfp)-FRT-kan-FRT*	This study
MMR182	MG1655 Δ(*lacI lacZYA*)::(P$_{emrK}$*-yfp)-FRT*	This study
MMR183	MG1655 *evgS1* Δ(*lacI lacZYA*)::(P$_{emrK}$*-yfp)-FRT*	This study
MMR191	MG1655 Δ*evgAS*::(FRT-kan-FRT) Δ(*lacI lacZYA*)::(P$_{emrK}$*-yfp)-FRT-kan-FRT*	This study
MMR227	Nissle 1917 Δ(*lacI lacZYA*)::(P$_{yfdX}$*-yfp)-FRT-kan-FRT*	This study
MMR228	UTI89 Δ(*lacI lacZYA*)::(P$_{yfdX}$*-yfp)-FRT-kan-FRT*	This study
MMR236	MG1655 *evgS1* Δ*torI*::(*FRT-kan-FRT*) Δ(*lacI lacZYA*)::(P$_{emrK}$*-yfp)-FRT-kan-FRT*	This study
MMR237	MG1655 Δ*torI*::(*FRT-kan-FRT*) Δ(*lacI lacZYA*)::(P$_{emrK}$*-yfp)*	This study
MMR239	MG1655 Δ*ydeO*::(*FRT-kan-FRT*) Δ(*lacI lacZYA*)::(P$_{hdeA}$*-yfp)*	This study
MMR241	MG1655 Δ*evgAS*::FRT *att*$_{\lambda}$::(P$_{mgrB}$*-yfp*)	This study
MMR254	Nissle 1917 *att*$_{\lambda}$::(P$_{mgrB}$*-yfp*)-kan	This study
MMR255	UTI89 *att*$_{\lambda}$::(P$_{mgrB}$*-yfp*)-kan	This study
MMR272	Nissle 1917 Δ(*lacI lacZYA*)::(P$_{hdeA}$*-yfp)-FRT-kan-FRT*	This study
MMR273	UTI89 Δ(*lacI lacZYA*)::(P$_{hdeA}$*-yfp)-FRT-kan-FRT*	This study
MP131	MP1 *att*$_{\lambda}$::(P$_{mgrB}$*-yfp*)	This study
MP136	MP1 Δ(*lacI lacZYA*)::(P$_{emrK}$*-yfp)-FRT-kan-FRT*	This study
MP137	MP1 *evgS1* Δ*torI*::*FRT* Δ(*lacI lacZYA*)::(P$_{emrK}$*-yfp)*	This study
MP138	MP1 Δ(*lacI lacZYA*)::(P$_{hdeA}$*-yfp)-FRT-kan-FRT*	This study

MP139	MP1 *evgS1* Δ*torI*::*FRT* Δ(*lacI lacZYA*)::(P_{hdeA}-*yfp)-FRT-kan-FRT*	This study
MP140	MP1 *evgS1* Δ*torI*::*FRTatt*$_{\lambda}$::(P_{mgrB}-*yfp*)	This study
MP142	MP1 *att*$_{\lambda}$::(P_{mgrB}-*yfp*)-kan	This study
MP143	MP1 *evgS1* Δ*torI*::*FRT* *att*$_{\lambda}$::(P_{mgrB}-*yfp*)-kan	This study
MP144 (MP1*)	MP1 *safA*$^{+}$*ydeO*$^{+}$ Δ*yneL*::(*FRT-kan-FRT*)	This study
MP145	MP1 *evg*SF577S (F577SEvgS$_{MP1}$) Δ(*lacI lacZYA*)::(P_{emrK}-*yfp)-FRT*	This study
MP146	MP1 Δ(*lacI lacZYA*)::(P_{emrK}-*yfp)-FRT*	This study
MP162	MP1 Δ*evgAS*::(FRT-kan-FRT) Δ(*lacI lacZYA*)::(P_{emrK}-*yfp)-FRT-kan-FRT*	This study
MP200	MP1 Δ*phoP*::FRT	This Study
JW1494	Δ*ydeO*::(*FRT-kan-FRT*)	(Baba, Ara et al. 2006)
JW5244	Δ*yneL*::(*FRT-kan-FRT*)	(Baba, Ara et al. 2006)
JW5387	Δ*torI*::(*FRT-kan-FRT*)	(Baba, Ara et al. 2006)
PIR2	*F*$^{-}$ *Δ lac169 rpoS*(*Am*) *robA1 creC510 hsdR514 endA recA1 uidA(ΔMluI)::pir*	Invitrogen
E. cloni	*F*$^{-}$ *mcrA Δ(mrr-hsdRMS-mcrBC) endA1 recA1 φ80lacZΔM15 ΔlacX74 araD139 Δ(ara-leu)7697 galU galK rpsL nupG tonA (attL araC*-P_{BAD}-*trfA250 bla attR) λ−*	Lucigen Corporation

References

Baba, T., T. Ara, et al. (2006). "Construction of Escherichia coli K-12 in-frame, single-gene knockout mutants: the Keio collection." Mol Syst Biol 2: 2006 0008.

Campellone, K. G., A. Giese, et al. (2002). "A tyrosine-phosphorylated 12-amino-acid sequence of enteropathogenic Escherichia coli Tir binds the host adaptor protein Nck and is required for Nck localization to actin pedestals." Mol Microbiol 43(5): 1227-1241.

Crossman, L. C., R. R. Chaudhuri, et al. (2010). "A commensal gone bad: complete genome sequence of the prototypical enterotoxigenic Escherichia coli strain H10407." J Bacteriol 192(21): 5822-5831.

Grozdanov, L., C. Raasch, et al. (2004). "Analysis of the genome structure of the nonpathogenic probiotic Escherichia coli strain Nissle 1917." J Bacteriol 186(16): 5432-5441.

Iguchi, A., N. R. Thomson, et al. (2009). "Complete genome sequence and comparative genome analysis of enteropathogenic Escherichia coli O127:H6 strain E2348/69." J Bacteriol 191(1): 347-354.

Lasaro, M., Z. Liu, et al. (2014). "Escherichia coli isolate for studying colonization of the mouse intestine and its application to two-component signaling knockouts." J Bacteriol 196(9): 1723-1732.
Luo, C., S. T. Walk, et al. (2011). "Genome sequencing of environmental Escherichia coli expands understanding of the ecology and speciation of the model bacterial species." Proc Natl Acad Sci U S A 108(17): 7200-7205.
Miyashiro, T. and M. Goulian (2007). "Stimulus-dependent differential regulation in the Escherichia coli PhoQ PhoP system." Proc Natl Acad Sci U S A 104(41): 16305-16310.
Mobley, H. L., D. M. Green, et al. (1990). "Pyelonephritogenic Escherichia coli and killing of cultured human renal proximal tubular epithelial cells: role of hemolysin in some strains." Infect Immun 58(5): 1281-1289.
Mulvey, M. A., J. D. Schilling, et al. (2001). "Establishment of a persistent Escherichia coli reservoir during the acute phase of a bladder infection." Infect Immun 69(7): 4572-4579.
Rasko, D. A., M. J. Rosovitz, et al. (2008). "The pangenome structure of Escherichia coli: comparative genomic analysis of E. coli commensal and pathogenic isolates." J Bacteriol 190(20): 6881-6893.
Walk, S. T., E. W. Alm, et al. (2009). "Cryptic lineages of the genus Escherichia." Appl Environ Microbiol 75(20): 6534-6544.
