# Supplementary material for: Natural variation of a sensor kinase controlling a conserved stress response pathway in Escherichia coli
Source: PLoS Genet. 2017 Nov 15;13(11):e1007101. doi: 10.1371/journal.pgen.1007101 (PMC5706723; doi:10.1371/journal.pgen.1007101)
Supplement: S2 Table — (PDF) [file pgen.1007101.s003.pdf]

S2 Table. List of plasmids used in this study.

Plasmid	Relevant Genotype	Reference or Source
pCP20	λ*cI857*(ts) *repA101*(ts) *oriR101 bla cat* λ*p*R-FLP	(Cherepanov and Wackernagel 1995)
EB45	pKD13 *yfp*-FRT-*kan*-FRT	(Batchelor and Goulian 2006)
pWRG99	pKD46 P_{tetA}-(I-*Sce*I *tetR*)	(Blank, Hensel et al. 2011)
pWRG100	pKD3 I-*Sce*I restriction site	(Blank, Hensel et al. 2011)
pSMART	pSMART VC BamHI (BAC) Cm^r *oriV ori2 repE IncC parABC*	Lucigen Corporation
P*phoP-gfp*		Open Biosystems
pMR69	pKD13 $P_{emrK\text{-MG1655}}$-*yfp*-FRT-*kan*-FRT	This study
pMR70	pKD13 $P_{emrK\text{-MP1}}$-*yfp*-FRT-*kan*-FRT	This study
pMR71	pKD13 $P_{hdeA\text{-MG1655}}$-*yfp*-FRT-*kan*-FRT	This study
pMR72	pKD13 $P_{hdeA\text{-MP1}}$-*yfp*-FRT-*kan*-FRT	This study
pMR73	pKD13 $P_{yfdX\text{-MG1655}}$-*yfp*-FRT-*kan*-FRT	This study
pMR76 (p*safAydeO*$_{MG1655}$)	pSMART *safAydeO*$_{MG1655}$	This study
pMR78 (p*evgAS*$_{MG1655}$)	pSMART *evgAS*$_{MG1655}$	This study
pMR80 (Hybrid A)	pSMART *evgA*$_{MG1655}$*evgS*$_A$(EvgS[537-1197]$_{MP1}$)	This study
pMR82 (Hybrid C)	pSMART *evgA*$_{MG1655}$*evgS*$_C$(EvgS[347-1197]$_{MP1}$)	This study
pMR83 (Hybrid D)	pSMART *evgA*$_{MG1655}$*evgS*$_D$(EvgS[347-537]$_{MP1}$)	This study
pMR84 (p*evgAS*$_{MP1}$)	pSMART *evgAS*$_{MP1}$	This study
pMR86	pSMART $P_{yfdX\text{-MG1655}}$-*yfp*	This study
pMR92 (Hybrid B)	pSMART *evgA*$_{MG1655}$*evgS*$_B$(EvgS[1-537]$_{MP1}$)	This study
pMR117 (*evgA*$_{MP1}$*evgS*$_{MG1655}$)	pSMART *evgA*$_{MP1}$*evgS*$_{MG1655}$	This study
pMR128 (p*evgAS*$_{MG1655}$-*safAydeO*$_{MG1655}$)	pSMART *evgAS*$_{MG1655}$-*safAydeO*$_{MG1655}$	This study

References

Batchelor, E. and M. Goulian (2006). "Imaging OmpR localization in Escherichia coli." Mol Microbiol 59(6): 1767-1778.

Blank, K., M. Hensel, et al. (2011). "Rapid and highly efficient method for scarless mutagenesis within the Salmonella enterica chromosome." PLoS One 6(1): e15763.

Cherepanov, P. P. and W. Wackernagel (1995). "Gene disruption in Escherichia coli: TcR and KmR cassettes with the option of Flp-catalyzed excision of the antibiotic-resistance determinant." Gene 158(1): 9-14.
