# Supplementary material for: Natural variation of a sensor kinase controlling a conserved stress response pathway in Escherichia coli
Source: PLoS Genet. 2017 Nov 15;13(11):e1007101. doi: 10.1371/journal.pgen.1007101 (PMC5706723; doi:10.1371/journal.pgen.1007101)
Supplement: S3 Table — (PDF) [file pgen.1007101.s004.pdf]

S3 Table. Primers used in this study.

Name	Sequence	Uses
hdeABprom-BamHI-U2	ccctgactggatcCGACTGCGACGAGGAAAC	Cloning of the P_{hdeA} –*yfp* reporter
hdeABprom-CFP-L1	ctcctctttaattctaggtacCACTGAGGTTATAACCTGGTTTTC	Cloning of the P_{hdeA} –*yfp* reporter
emrK-BamHI-U1	cctgactggatccTTCCGCCTTCAGTCAACTC	Cloning of the P_{emrK} –*yfp* reporter
emrK-KpnI-L1	cctgactggtaccGCACATTATCCCAGGAACG	Cloning of the P_{emrK} –*yfp* reporter
yfdX-BamHI-U1	cctgactggatccGACGCTATCGCACAGGATG	Cloning of the P_{yfdX} –*yfp* reporter
yfdX-KpnI-L1	cctgactggtaccAAACGCCGCCATCATATTGGG	Cloning of the P_{yfdX} –*yfp* reporter
lac-pMR10-U1	*GGTGGCCGGAAGGCGAAGCGGCATGCATTTACGTTGACACCATCGAATGG*GCAATCCAGTGCAAAGCTAG	Integration of P_{hdeA} –*yfp*, P_{emrK} –*yfp*, P_{yfdX} –*yfp* reporters
lac-pMR10-L1	*GCCTTCGCACATATCGGTAAATAGCTTGCCTGCTCTTATTCTTTCGG*GAGCGATTGTGTAGGCTGGAG	Integration of P_{hdeA} –*yfp*, P_{emrK} –*yfp*, P_{yfdX} –*yfp* reporters
evgS-F577-For	*CGCTCAGTTCGTCGTCGTAAAGTCATTCAGGGTGATTTAGAAAACCAAATA*CGCCTTACGCCCCGCCCTGC	Generation of a Cm-I-SceI cassette with flanking homology to *evgS*
evgS-F577-Rev	*CCTTGCCAGTTTACAACATAAGTTGGATTCGGTAAGGAATCCGAGAGTGCT*CTAGACTATATTACCCTGTT	Generation of a Cm-I-SceI cassette with flanking homology to *evgS*
F577S-For	TAGAAAACCAAATATCAT*C*CCGAAAAGCACTCTCGG	Generation of EvgS-F577S$_{MG1655}$
F577S-Rev	CCGAGAGTGCTTTTCGG*G*ATGATATTTGGTTTTCTA	Generation of EvgS-F577S$_{MG1655}$
evgS-F	CGCGGAGAACCGGAACTTAAGG	Generation of EvgS-F577S$_{MG1655}$
evgS-R	GCAGGAAGATTGCATAATGTATGCCAGTG	Generation of EvgS-F577S$_{MG1655}$
torIDel-left	CTCACACTCGATGAGGCCTG	Generation of Δ*torI*::(*FRT-kan-FRT*)
torIDel-right	GCAGACATCTACTTGAAGCAGC	Generation of Δ*torI*::(*FRT-kan-FRT*)
evgA-Nterm-lred-u1	ATTATTGATGACCATCCTCTTGCTATCGCAATTCCGGGGATCCGTCGACC	Generation of Δ*evgAS*::(*FRT-kan-FRT*)
evgS-Cterm-lred-l2	TCTGAAACAGGTGTTATTTCTAACTGATGGTGTAGGCTGGAGCTGCTTCG	Generation of Δ*evgAS*::(*FRT-kan-FRT*)
b1500-red-u1	ACTGATTAACGATTTTTAACGTTATCCGCTAAATAAACATATTTGAAATGATTCCGGGGATCCGTCGACC	Generation of Δ*safA*::(*FRT-kan-FRT*)
b1500-red-l1	ATTTCATATTTATAATTTGCTGTTTGTTTTCAGCCTTGCAAACTATTGATTGTAGGCTGGAGCTGCTTCG	Generation of Δ*safA*::(*FRT-kan-FRT*)
evgA-EcoRI-U1	ccgacacgaattcGCACATTATCCCAGGAACGTTGGAG	Cloning of *evgAS*

evgS-BamHI-L1	ccgacacggatccCATTGTGGGAGCCGCTATTTAGTC	Cloning of *evgAS*
CFP-EcoRI-l1	cccgaattcGTCGACCAGGTCAGCTAATTAAG	Construction of P_{yfdX} –*yfp* plasmid reporter
safA-BamHI-U1	ccctgacggatccCCACGATCCATTAAGTGGCATTCC	Cloning of *safAydeO*
ydeO-HindIII-L1	ccctgacaagcttCAGTCCATAAAGCGTTGACATTAC	Cloning of *safAydeO*
tL3 term-l1	gggatccTTCTTCGTCTGTTTCTACTG	Construction of P_{yfdX} –*yfp* plasmid reporter
MP1-F577S-For	CGTAAAGTTATTCAGGGTGATTTAGAAAACCAAATATCA*TCG*CGGAAAGC	Generation of EvgS-F577S$_{MP1}$
MP1-Tn5-evgS-R	GCTTGCGGCAGCGTGAGCTTCAAAAGCGCTCTGGTGGGAGCCGCTATTTAGTCATTTTGCTGACAG	Generation of EvgS-F577S$_{MP1}$
MP1-evgS-Tn5-F	CTGTCAGCAAAATGACTAAATAGCGGCTCCCACCAGAGCGCTTTTGAAGCTCACGCTGCCGCAAGC	Generation of EvgS-F577S$_{MP1}$
MP1-Tn5-end-yfdE	GCCAGGAGTTCTCATCATAAATGTAAGATGTGCTGGTAAATAGCTCCCACGATCCCCTTATTAGAAGAACTCGTC	Generation of EvgS-F577S$_{MP1}$
evgS-swap5	ACGACGAACTGAGCGTAAC	Generation of Hybrid EvgS(538-1197)$_{MP1}$
evgS-swap3	GTGTGAAACGGTGATCGGAG	Generation of Hybrid EvgS(347-1197)$_{MP1}$
evgS-swap4	GGGCGTTATGGGGGACATTC	Generation of Hybrid EvgS(347-1197)$_{MP1}$
evgA-evgS-F	GGATCTTTACACATTCGCACAAC	Generation of p*evgA*$_{MP1}$*evgS*$_{MG1655}$
evgA-evgS-R	GTCGACCAAAGACCACAACAG	Generation of p*evgA*$_{MP1}$*evgS*$_{MG1655}$

Sequences added for cloning purpose are in lower case.
Restriction sites are in underlined lower case.
Flanking sequences of homology for integration in a chromosomal site are italicized.
Underlined and italicized indicates nucleotide mutations.
