# Supplementary material for: Natural variation of a sensor kinase controlling a conserved stress response pathway in Escherichia coli
Source: PLoS Genet. 2017 Nov 15;13(11):e1007101. doi: 10.1371/journal.pgen.1007101 (PMC5706723; doi:10.1371/journal.pgen.1007101)
Supplement: S4 Table — (PDF) [file pgen.1007101.s005.pdf]

S4 Table. Sequence evolution of *evgS* based on dN/dS analysis.

	All 10 *E. coli* isolates			Group A			Group B2		
**Gene**	**dN**[a]	**dS**[b]	**dN/dS**[c]	**dN**	**dS**	**dN/dS**	**dN**	**dS**	**dN/dS**
evgS	0.0116	0.1143	0.1013	0.0016[d], 0.0006[e]	0.0051[d], 0.0014[e]	0.3240[d], 0.4409[e]	0.0009[f], 0.0011[g]	0.0116[f], 0.0152[g]	0.0802[f], 0.0744[g]
evgS (1-537)	0.0136	0.1232	0.1101	0.0027	0.0114	0.2328	0.0006	0.0057	0.1131
evgS (87-234)	0.0146	0.1269	0.1149	0.0059	0.0131	0.4529	0	0	ND[h]
evgA	0	0.0609	0	0	0	ND[h]	0	0.0079	0
yfdE	0.0064	0.0965	0.0658	0	0.0105	0	0.0043	0.0498	0.0856
barA	0.0004	0.0671	0.0056	0	0.0031	0	0.0004	0.0388	0.0097
arcB	0.0008	0.0627	0.0134	0.0004	0	ND[h]	0.0003	0.0139	0.0239

[a]dN – Average number of substitutions per non-synonymous site

[b]dS – Average number of substitutions per synonymous site

[c]dN/dS – Ratio of rates of non-synonymous (dN) and synonymous (dS) substitutions; also known as Ka/Ks

[d,f]values corresponding to the *E. coli* strains included in this study belonging to phylogenetic groups A and group B2, respectively

[e,g]values corresponding to all the *E. coli* strains from phylogenetic groups A and B2, respectively, derived from the MLST-based tree (Lasaro, Liu et al. 2014)

[h]ND – Not determined

Lasaro, M., Z. Liu, et al. (2014). "Escherichia coli isolate for studying colonization of the mouse intestine and its application to two-component signaling knockouts." J Bacteriol **196**(9): 1723-1732.
