# Supplementary material for: Natural variation of a sensor kinase controlling a conserved stress response pathway in Escherichia coli
Source: PLoS Genet. 2017 Nov 15;13(11):e1007101. doi: 10.1371/journal.pgen.1007101 (PMC5706723; doi:10.1371/journal.pgen.1007101)
Supplement: S5 Table — (PDF) [file pgen.1007101.s006.pdf]

S5 Table. d*N*/d*S* analysis for *evgS* (*Escherichia coli*), *bvgS* (*Bordetella pertussis*), *kvgS* (*Klebsiella pneumoniae*) and related genes based on currently available complete genomes at NCBI.

Gene	d*N*/d*S*[c]	d*N*[a]	d*S*[b]
Escherichia coli			
evgS	0.11	0.0085	0.0771
evgA	0.01	0.0002	0.0391
yfdE	0.07	0.0048	0.0638
barA	0.01	0.0004	0.0479
arcB	0.01	0.0004	0.0409
Bordetella pertussis			
bvgS	ND[d]	0.0000	0.0000
bvgA	0.00	0.0000	0.0001
Klebsiella pneumoniae			
kvgS	0.13	0.0036	0.0278
kvgA	0.14	0.0026	0.0186
yfdX[e]	0.10	0.0007	0.0069
barA	0.03	0.0011	0.0316
arcB	0.01	0.0003	0.0358

[a]d*N* – Average number of substitutions per non-synonymous site
[b]d*S* – Average number of substitutions per synonymous site
[c]d*N*/d*S* – Ratio of rates of non-synonymous (d*N*) and synonymous (d*S*) substitutions, also known as Ka/Ks
[d]ND – not determined; dN and dS are both 0.
[e]*yfdX* is a neighboring gene for *kvgS* in *K. pneumoniae*; yfdE is missing in this organism
