# Supplementary material for: Natural variation of a sensor kinase controlling a conserved stress response pathway in Escherichia coli
Source: PLoS Genet. 2017 Nov 15;13(11):e1007101. doi: 10.1371/journal.pgen.1007101 (PMC5706723; doi:10.1371/journal.pgen.1007101)
Supplement: S1 Fig — A: The PhoQ/PhoP system is not activated by mild acidity over a range of pH values. Transcriptional reporter derivatives of MG1655 (TIM92), MG1655 ΔsafA (SAM74), and MP1 (MP131) were cultured to OD600 ~0.2 in minimal medium containing 10 mM MgSO4 and buffered with 100 mM MES, at pH 5.1, 5.3, 5.5, 5.7, 5.9, 6.1, 6.3, and 7. Fluorescence was determined as described in Materials and methods. Fluorescence values are the average from two independent experiments. Error bars represent the range. B: mgrB is activated in MP1 by low Mg++ similarly to E. coli K-12. Strains TIM63 (MG1655 PmgrB-yfp) and MP131 (MP1 PmgrB-yfp) were cultured to OD600 ~0.2 in minimal medium at pH 7 with either 10 mM or 1 μm MgSO4. Fluorescence was determined as described in Materials and methods. Fluorescence values are the average from two independent experiments. Error bars represent the range. (PDF) [file pgen.1007101.s007.pdf]

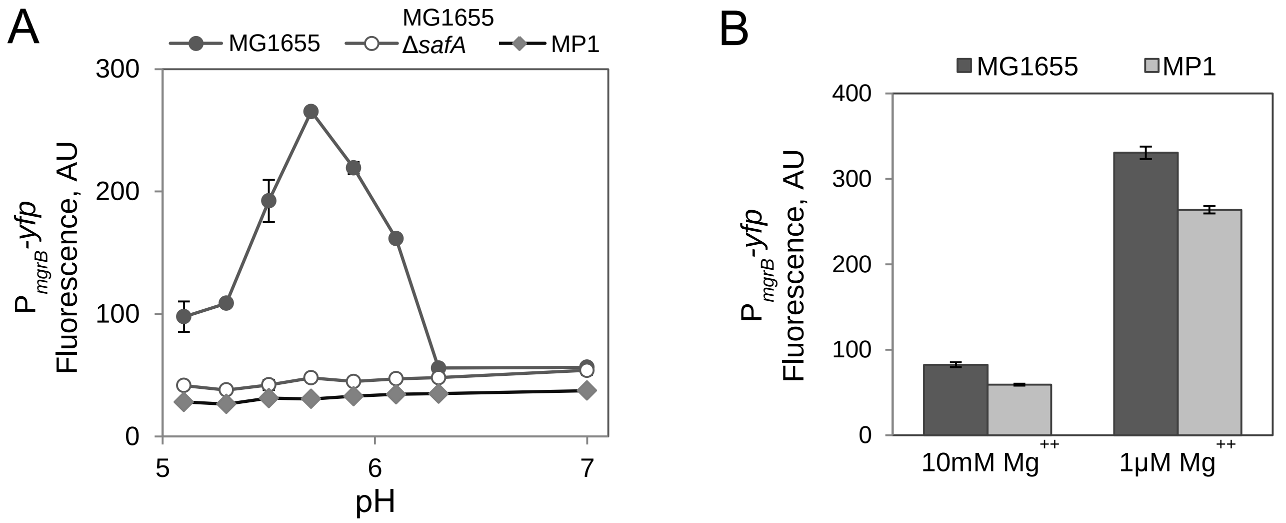

S1 Fig. Activation of the PhoQ/PhoP system from different stimuli. A: The PhoQ/PhoP system is not activated by mild acidity over a range of pH values**.** Transcriptional reporter derivatives of MG1655 (TIM92), MG1655 Δ*safA* (SAM74), and MP1 (MP131) were cultured to OD_{600} ~0.2 in minimal medium containing 10 mM $MgSO_4$ and buffered with 10 mM MES, at pH 5.1, 5.3, 5.5, 5.7, 5.9, 6.1, 6.3, and 7. Fluorescence was determined as described in Materials and methods. Fluorescence values are the average from two independent experiments. Error bars represent the range. B: *mgrB* is activated in MP1 by low Mg^{++} similarly to *E. coli* K-12. Strains TIM63 (MG1655 P_{mgrB}-*yfp)* and MP131 (MP1 P_{mgrB}-*yfp*) were cultured to OD_{600} ~0.2 in minimal medium at pH 7 with either 10 mM or 1 μm $MgSO_4$. Fluorescence was determined as described in Materials and methods. Fluorescence values are the average from two independent experiments. Error bars represent the range.
