# Supplementary material for: Natural variation of a sensor kinase controlling a conserved stress response pathway in Escherichia coli
Source: PLoS Genet. 2017 Nov 15;13(11):e1007101. doi: 10.1371/journal.pgen.1007101 (PMC5706723; doi:10.1371/journal.pgen.1007101)
Supplement: S2 Fig — A: resistance of strains MG1655 and MP1 in different growth stages. Strains were cultured in minimal medium at pH 7 to stationary phase (16 hours) and at pH 5.7 to exponential phase (OD600 ~0.2). Cultures were shocked for an hour at pH 2.5 as described in Materials and methods. Values are the average percent survival from two representative experiments and error bars represent the range. B: Resistance of MP1*, a strain derived from MP1 by transducing from MG1655 a region encompassing the 13 kb segment that is absent in MP1. Strains MG1655, MP1 and MP1* (MP144) were cultured at pH 5.7 to exponential phase (OD600 ~0.2), and acid resistance was assayed as in A. Values are the average percent survival from two representative experiments and error bars represent the range. C: Strains MG1655, MMR241 (MG1655 ΔevgAS), TIM96 (MG1655 ΔphoQP), and SAM74 (MG1655 ΔsafA) were cultured and assayed for acid resistance as in B, except samples were withdrawn at the indicated times. Values are the average percent survival from two representative experiments and error bars represent the range. (PDF) [file pgen.1007101.s008.pdf]

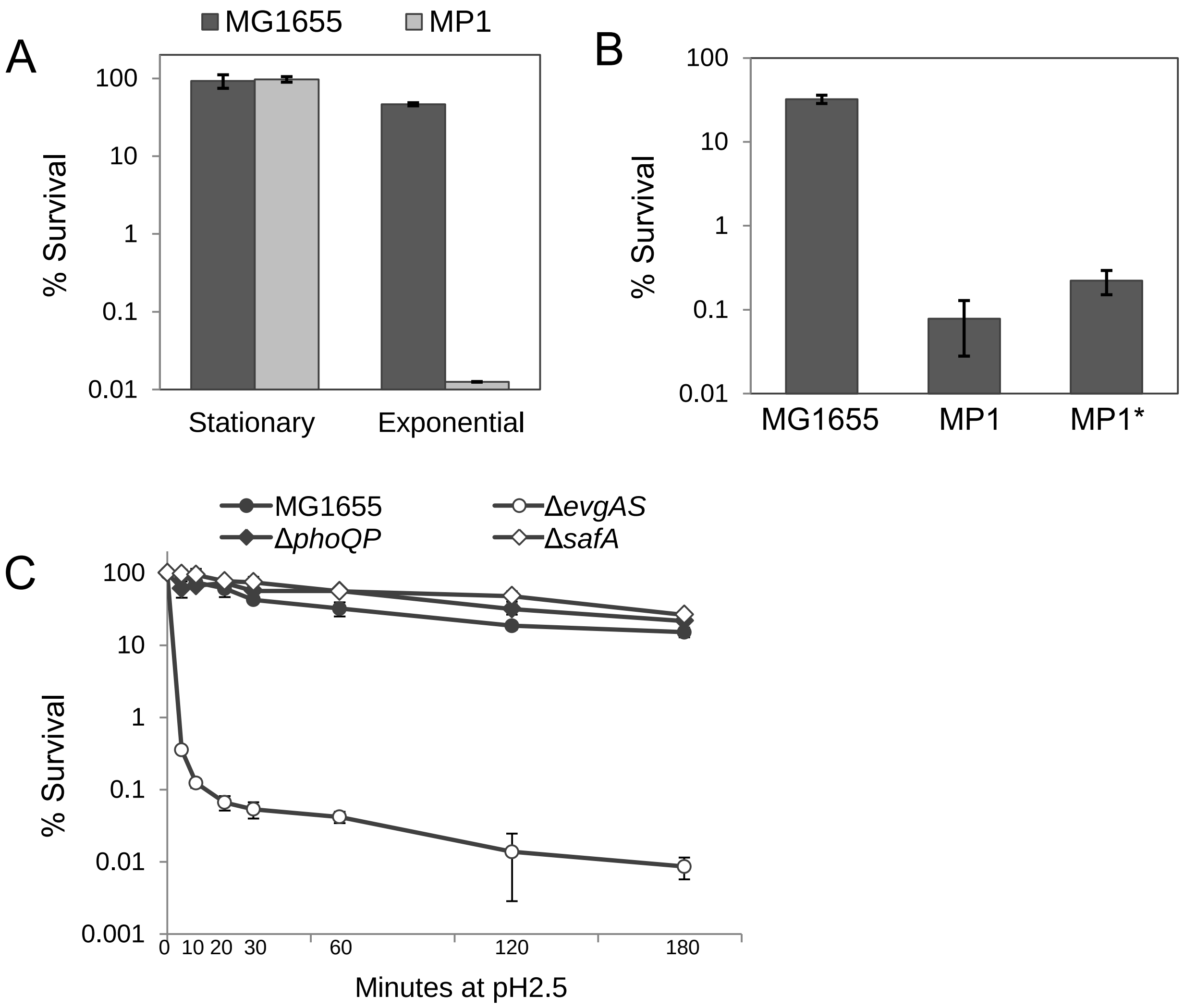

S2 Fig. Resistance to acid shock. A: resistance of strains MG1655 and MP1 in different growth stages. Strains were cultured in minimal medium at pH7 to stationary phase (16 hours) and at pH 5.7 to exponential phase (OD_{600} ~0.2). Cultures were shocked for an hour at pH 2.5 as described in Materials and methods. Values are the average percent survival from two representative experiments and error bars represent the range. B: Resistance of MP1*, a strain derived from MP1 by transducing from MG1655 a region encompassing the 13 kb segment that is absent in MP1. Strains MG1655, MP1 and MP1* (MP144) were cultured at pH 5.7 to exponential phase (OD_{600} ~0.2), and acid resistance was assayed as in A. Values are the average percent survival from two representative experiments and error bars represent the range. C: Strains MG1655, MMR241 (MG1655 Δ*evgAS*), TIM96 (MG1655 Δ*phoQP*), and SAM74 (MG1655 Δ*safA*) were cultured and assayed for acid resistance as in B, except samples were withdrawn at the indicated times. Values are the average percent survival from two representative experiments and error bars represent the range.
