# Supplementary material for: Natural variation of a sensor kinase controlling a conserved stress response pathway in Escherichia coli
Source: PLoS Genet. 2017 Nov 15;13(11):e1007101. doi: 10.1371/journal.pgen.1007101 (PMC5706723; doi:10.1371/journal.pgen.1007101)
Supplement: S3 Fig — Strains MMR182 (MG1655 PemrK-yfp) and MP146 (MP1 PemrK-yfp) were cultured in minimal medium at pH 7 or in minimal medium buffered with 100 mM MES at pH 5.1, 5.3, 5.5, 5.7, 5.9, 6.1, 6.3, and 7. Cultures were harvested at OD600~0.2, and fluorescence of the reporter was measured as described in Materials and methods. Fluorescence values are the average from two representative independent experiments. Error bars represent the range. (PDF) [file pgen.1007101.s009.pdf]

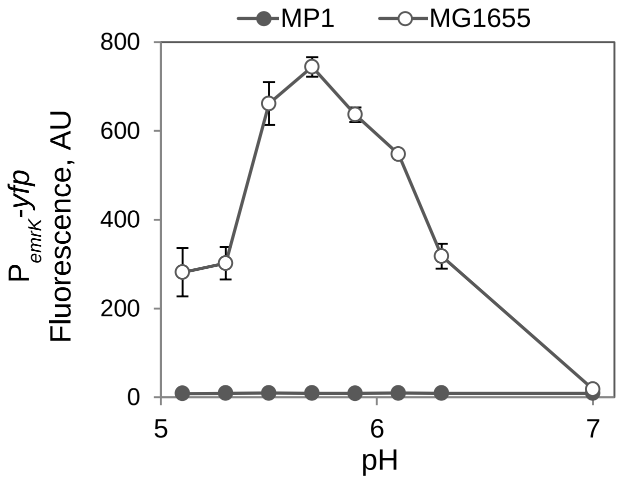

S3 Fig. $EvgS_{MP1}$ is not responsive to mild acidity in the pH range effective for $EvgS_{MG1655}$. Strains MMR182 (MG1655 P_{emrK}-*yfp)* and MP146 (MP1 P_{emrK}-*yfp*) were cultured in minimal medium at pH7 or in minimal medium buffered with 100 mM MES at pH 5.1, 5.3, 5.5, 5.7, 5.9, 6.1, 6.3, and 7. Cultures were harvested at OD_{600}~0.2, and fluorescence of the reporter was measured as described in Materials and methods. Fluorescence values are the average from two representative independent experiments. Error bars represent the range.
