# Supplementary material for: Natural variation of a sensor kinase controlling a conserved stress response pathway in Escherichia coli
Source: PLoS Genet. 2017 Nov 15;13(11):e1007101. doi: 10.1371/journal.pgen.1007101 (PMC5706723; doi:10.1371/journal.pgen.1007101)
Supplement: S4 Fig — A: Diagram illustrating the plasmids and hybrid EvgS proteins utilized in this assay. Predicted transmembrane domains (TM), and residues they span, are indicated. (*) indicates a transmembrane domain that is predicted in some databases (www.uniprot.org) but is inconsistent with a homology model based on the ortholog BvgS [11]. Residues delimiting the swapped EvgS regions are numbered at the bottom and indicated by the dotted lines. B: Activity of the EvgA-dependent reporter PemrK-yfp. The hybrid names correspond to those indicated in panel A. Strains with either wild type evgAS (MMR182) carrying the empty vector (pSMART), or ΔevgAS (MMR191) with the empty vector, or one of the plasmids pMR78, pMR117, pMR84, pMR80, pMR92, pMR82, pMR83 (in this order in the figure) were cultured in minimal medium at pH 7 and pH 5.7 to OD600~0.2. Fluorescence was quantified by microscopy as described in Materials and methods. Values are the average fluorescence from two independent experiments and error bars represent the range. (PDF) [file pgen.1007101.s010.pdf]

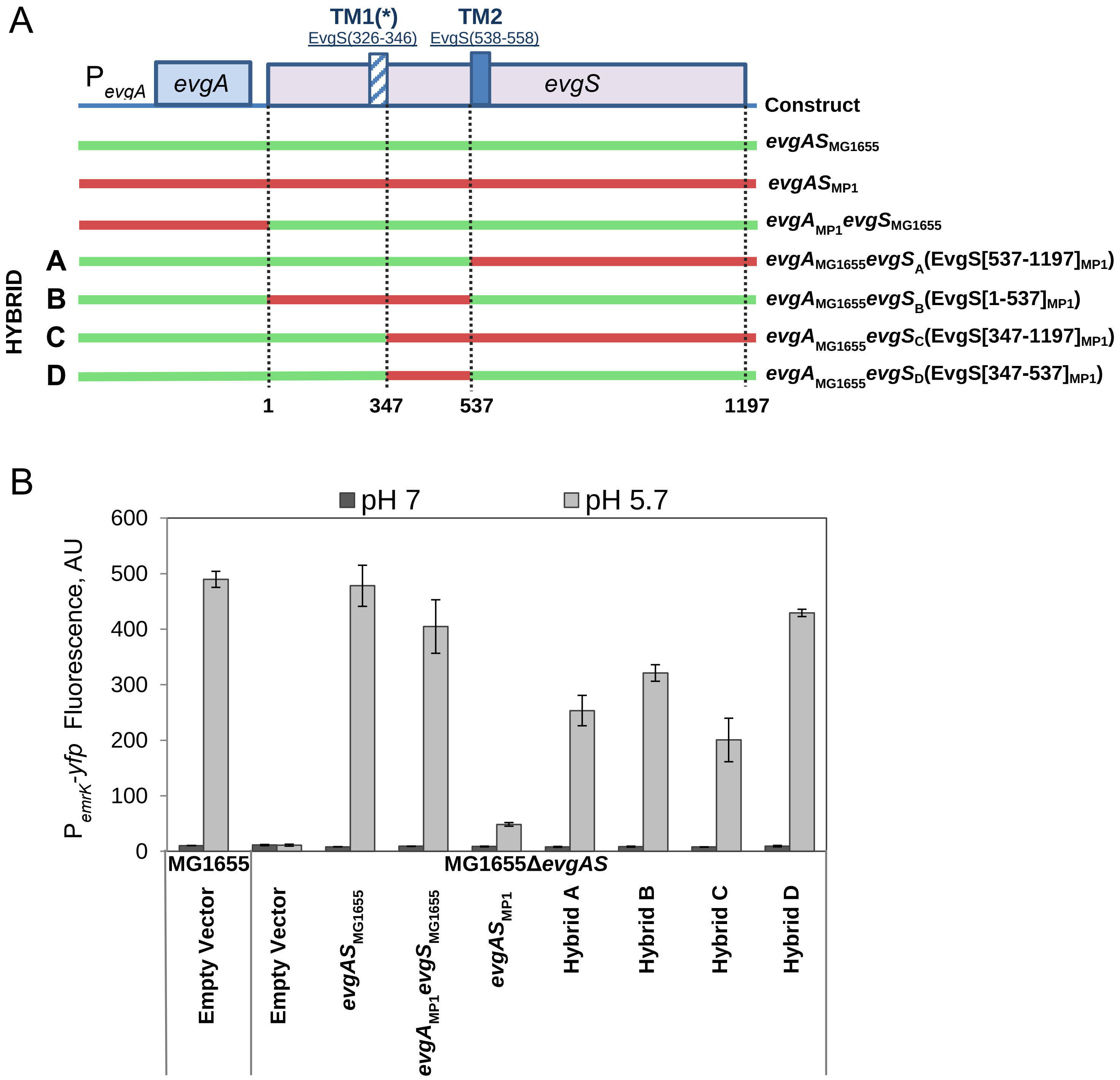

S4 Fig. EvgS domain swap between MG1655 and MP1. A: Diagram illustrating the plasmids and hybrid EvgS proteins utilized in this assay. Predicted transmembrane domains (TM), and residues they span, are indicated. (*) indicates a transmembrane domain that is predicted in some databases (www.uniprot.org) but is inconsistent with a homology model based on the ortholog BvgS [11]. Residues delimiting the swapped EvgS regions are numbered at the bottom and indicated by the dotted lines. B: Activity of the EvgA-dependent reporter P_{emrK}-*yfp*. The hybrid names correspond to those indicated in panel A. Strains with either wild type *evgAS* (MMR182) carrying the empty vector (pSMART), or Δ*evgAS* (MMR191) with the empty vector, or one of the plasmids pMR78, pMR117, pMR84, pMR80, pMR92, pMR82, pMR83 (in this order in the figure) were cultured in minimal medium at pH 7 and pH 5.7 to OD_{600}~0.2. Fluorescence was quantified by microscopy as described in Materials and methods. Values are the average fluorescence from two independent experiments and error bars represent the range.
