# Supplementary material for: Natural variation of a sensor kinase controlling a conserved stress response pathway in Escherichia coli
Source: PLoS Genet. 2017 Nov 15;13(11):e1007101. doi: 10.1371/journal.pgen.1007101 (PMC5706723; doi:10.1371/journal.pgen.1007101)
Supplement: S5 Fig — Derivatives of MG1655 and MP1 with the PemrK-yfp reporter and with WT evgS (MMR182 and MP146) or with the the reporter and the F577S amino acid substitution associated with the evgS1 allele (MMR183 and MP145) were cultured in minimal medium at pH 5.7 (induced wild-type strains) and pH 7 (F577SEvgS) strains and non-induced wild-type strains) to OD600~0.2 and fluorescence of the reporter was measured as described in Materials and methods. Fluorescence values are the average from two representative independent experiments. Error bars represent the range. (PDF) [file pgen.1007101.s011.pdf]

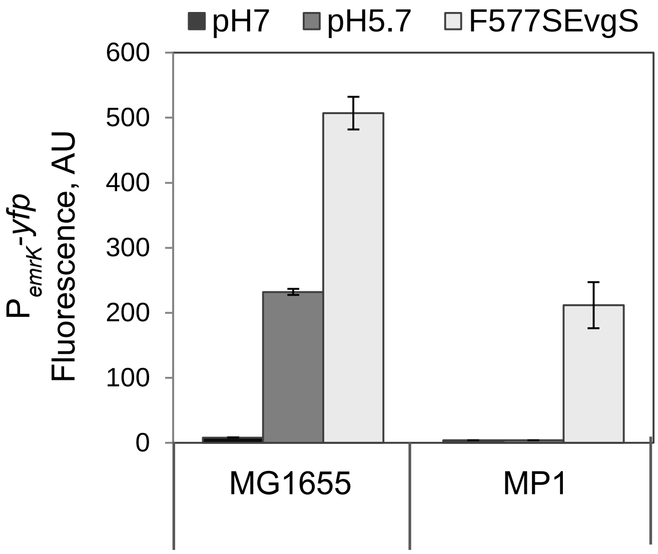

S5 Fig. The single residue change that makes EvgS$_{MG1655}$ constiutively active has a similar effect on EvgS$_{MP1}$. Derivatives of MG1655 and MP1 with the P$_{emrK}$-*yfp* reporter and with WT *evgS* (MMR182 and MP146) or with the the reporter and the F577S amino acid substitution associated with the *evgS1* allele (MMR183 and MP145) were cultured in minimal medium at pH5.7 (induced wild-type strains) and pH7 (F577SEvgS) strains and non-induced wild-type strains) to OD_{600}~0.2 and fluorescence of the reporter was measured as described in Materials and methods. Fluorescence values are the average from two representative independent experiments. Error bars represent the range.
