# Supplementary material for: Natural variation of a sensor kinase controlling a conserved stress response pathway in Escherichia coli
Source: PLoS Genet. 2017 Nov 15;13(11):e1007101. doi: 10.1371/journal.pgen.1007101 (PMC5706723; doi:10.1371/journal.pgen.1007101)
Supplement: S6 Fig — EvgS protein sequences from the indicated ten E. coli isolates were aligned as described in Materials and methods. (*) indicates a predicted transmembrane domain (www.uniProt.org) that is inconsistent with a homology model based on the ortholog BvgS [11]. The predicted PAS domain is shaded gray, and the green highlights indicate residues identified in previous studies as being involved in EvgS activity [4, 9–11]. (PDF) [file pgen.1007101.s012.pdf]

```
MG1655       1 MKFLPYIFLLCCGLWSTISFADEDYIEYRGISSNNRVTLDPLRLSNKELRWLASKKNLVI
HS           1 ............................................................
H10407       1 ............A....M..........................................
TW10509      1 ....T.F...S.C...............................................
EDL933       1 ............................................................
E2348/69     1 ......................G.....................................
CFT073       1 ...........X..........G.....H...............................
Nissle       1 ......................G.....................................
UTI89        1 ......................G.....................................
MP1          1 ......................G.....................................
consensus    1 ****.*.***...****.****.*****.*******************************

MG1655      61 AVHKSQTATLLHTDSQQRVRGINADYLNLLKRALNIKLTLREYADHQKAMDALAEGEVDI
HS          61 ............................................................
H10407      61 .....................................................E......
TW10509     61 .....K.........................K...........PN........E......
EDL933      61 .....................................................E......
E2348/69    61 ..................I..................................ED.....
CFT073      61 ..................I..................................ED.....
Nissle      61 ..................I..................................ED.....
UTI89       61 ..................I..................................ED.....
MP1         61 ..................I..................................ED.....
consensus   61 *****.************.************.***********..********..*****

                                               152
MG1655     121 VLSHLVTSPPLNNDIAATKPLIITFPALVTTLHDSMRPLTSPKPVNIARVANYPPDEVIH
HS         121 ............................................................
H10407     121 ......A.....D...............................................
TW10509    121 ......A...V.E...............................................
EDL933     121 ......A.....D............................S..................
E2348/69   121 ......A.....D.....N......................S..................
CFT073     121 ......A.....D.....N......................S..................
Nissle     121 ......A.....D.....N......................S..................
UTI89      121 ......A.....D.....N......................S..................
MP1        121 ......A.....D.....N......................S..................
consensus  121 ******.***.*.*****.**********************.******************

                                                             226
MG1655     181 QSFPKATIISFTNLYQALASVSAGHNDYFIGSNIITSSMISRYFTHSLNVVKYYNSPRQY
HS         181 ............................................................
H10407     181 ............................................................
TW10509    181 ......................T......M.....................N..ST....
EDL933     181 ........................Q...................................
E2348/69   181 ........................Q...................................
CFT073     181 ........................Q...................................
Nissle     181 ........................Q...................................
UTI89      181 ........................Q...................................
MP1        181 ........................Q...................................
consensus  181 **********************.*.****.*********************.**..****

MG1655     241 NFFLTRKESVILNEVLNRFVDALTNEVRYEVSQNWLDTGNLAFLNKPLELTEHEKQWIKQ
HS         241 ............................................................
H10407     241 ............................................................
TW10509    241 ..........V............................................L....
EDL933     241 ............................................................
E2348/69   241 ..L....D.IV.................................................
CFT073     241 ..L....D.IV.................................................
Nissle     241 ..L....D.IV.................................................
UTI89      241 ..L....D.IV.................................................
MP1        241 ..L....D.IV.................................................
consensus  241 **.****.*..********************************************.****
```

```
                                       Predicted Transmembrane Domain(*)
MG1655     301 HPNLKVLENPYSPPYSMTDENGSVRGVMGDILNIITLQTGLNFSPITVSHNIHAGTQLSP
HS         301 ............................................................
H10407     301 ............................................................
TW10509    301 ..E.R....................................K................N.
EDL933     301 ..D.......................................................N.
E2348/69   301 ..D.................T.....................................N.
CFT073     301 ..D.................T.....................................N.
Nissle     301 ..D.................T.....................................N.
UTI89      301 ..D.................T.....................................N.
MP1        301 ..D.................T.....................................N.
consensus  301 **.*.***************.********************.****************.*

                             375
MG1655     361 GGWDIIPGAIYSEDRENNVLFAEAFITTPYVFVMQKAPDSEQTLKKGMKVAIPYYYELHS
HS         361 ............................................................
H10407     361 ............................................................
TW10509    361 D....L......................................................
EDL933     361 .....L......................................................
E2348/69   361 .....L.A...........S.S.V....................................
CFT073     361 .....L.A...........S...V....................................
Nissle     361 .....L.A...........S...V....................................
UTI89      361 .....L.A...........S...V....................................
MP1        361 .....L.A...........S...V....................................
consensus  361 .****.*.***********.*.*.************************************

MG1655     421 QLKEMYPEVEWIQVDNASAAFHKVKEGELDALVATQLNSRYMIDHYYPNELYHFLIPGVP
HS         421 ............................................................
H10407     421 ............................................................
TW10509    421 ...........................................................E
EDL933     421 ............K...............................................
E2348/69   421 ............K..............................................Q
CFT073     421 ............K..............................................Q
Nissle     421 ............K..............................................Q
UTI89      421 ............K..............................................Q
MP1        421 ............K..............................................Q
consensus  421 ************.**********************************************.

                                                         522
MG1655     481 NASLSFAFPRGEPELKDIINKALNAIPPSEVLRLTEKWIKMPNVTIDTWDLYSEQFYIVT
HS         481 ............................................................
H10407     481 ............................................................
TW10509    481 ............................................................
EDL933     481 ............................................................
E2348/69   481 ............................................................
CFT073     481 ............................................................
Nissle     481 ............................................................
UTI89      481 ............................................................
MP1        481 ............................................................
consensus  481 ************************************************************

            Predicted Transmembrane Domain       573 574 577 582 584        600
MG1655     541 TLSVLLVGSSLLWGFYLLRSVRRRKVIQGDLENQISFRKALSDSLPNPTYVVNWQGNVIS
HS         541 ............................................................
H10407     541 ...........................................................
TW10509    541 ............................................................
EDL933     541 ............................................................
E2348/69   541 ............................................................
CFT073     541 ............................................................
Nissle     541 ............................................................
UTI89      541 ............................................................
MP1        541 ............................................................
consensus  541 ************************************************************
```

```
                                                          643            658
MG1655     601 HNSAFEHYFTADYYKNAMLPLENSDSPFKDVFSNAHEVTAETKENRTIYTQVFEIDNGIE
HS         601 ..........V.................................................
H10407     601 ............................................................
TW10509    601 ..................................V..............S.....N....
EDL933     601 ........................E.........T.........................
E2348/69   601 ..........D.............E.........T.........................
CFT073     601 ........................E.........T.........................
Nissle     601 ........................E.........T.........................
UTI89      601 ..........D.............E.........T.........................
MP1        601 ..........D.............E.........T.........................
consensus  601 **********.*************.*********.**************.*****.****

                         671                           701
MG1655     661 KRCINHWHTLCNLPASDNAVYICGWQDITETRDLINALEVEKNKAIKATVAKSQFLATMS
HS         661 ............................................................
H10407     661 ............................................................
TW10509    661 ...................................H.....R....N.............
EDL933     661 ................EH.................H.....R....N.............
E2348/69   661 ................EH.................H.....R....N.............
CFT073     661 ................EH.................H.....R....N.............
Nissle     661 ................EH.................H.....R....N.............
UTI89      661 ................EH.................H.....R....N.............
MP1        661 ................EH.................H.....R....N.............
consensus  661 ****************..*****************.*****.****.*************

               Histidine - phosphorylation site
MG1655     721 HEIRTPISSIMGFLELLSGSGLSKEQRVEAISLAYATGQSLLGLIGEILDVDKIESGNYQ
HS         721 ............................................................
H10407     721 ............................................................
TW10509    721 ............................................................
EDL933     721 ............................................................
E2348/69   721 ............................................................
CFT073     721 ............................................................
Nissle     721 ............................................................
UTI89      721 ............................................................
MP1        721 ............................................................
consensus  721 ************************************************************

MG1655     781 LQPQWVDIPTLVQNTCHSFGAIAASKSIALSCSSTFPEHYLVKIDPQAFKQVLSNLLSNA
HS         781 ............................................................
H10407     781 ............................................................
TW10509    781 .........A....................N....L..R.....................
EDL933     781 .....................................D......................
E2348/69   781 ...................A...............L..R.....................
CFT073     781 ...................A...............L..R.....................
Nissle     781 ...................A...............L..R.....................
UTI89      781 ...................A...............L..R.....................
MP1        781 ...................A...............L..R.....................
consensus  781 *********.*********.**********.****.*..*********************

MG1655     841 LKFTTEGAVKITTSLGHIDDNHAVIKMTIMDSGSGLSQEEQQQLFKRYSQTSAGRQQTGS
HS         841 ............................................................
H10407     841 ............................................................
TW10509    841 ...............V...............T...................T........
EDL933     841 ...............V............................................
E2348/69   841 ..................N.........................................
CFT073     841 ............................................................
Nissle     841 ............................................................
UTI89      841 ............................................................
MP1        841 ............................................................
consensus  841 ***************.**.************.*******************.********
```

```
MG1655     901 GLGLMICKELIKNMQGDLSLESHPGIGTTFTITIPVEISQQVATVEAKAEQPITLPEKLS
HS         901 ............................................................
H10407     901 ............................................................
TW10509    901 ......................................T.........P...V......N
EDL933     901 ......................................I....A................
E2348/69   901 ............................I.........T....A................
CFT073     901 ............................I.........T....A................
Nissle     901 ............................I.........T....A................
UTI89      901 ............................I.........T....A................
MP1        901 ............................I.........T.....................
consensus  901 ****************************.*********.****.****.***.******.
```

Aspartate – phosphorylation site

```
MG1655     961 ILIADDHPTNRLLLKRQLNLLGYDVDEATDGVQALHKVSMQHYDLLITDVNMPNMDGFEL
HS         961 ............................................................
H10407     961 ............................................................
TW10509    961 ............................S................F..............
EDL933     961 ......................................................V.....
E2348/69   961 ............................................................
CFT073     961 ............................................................
Nissle     961 ............................................................
UTI89      961 ............................................................
MP1        961 ............................................................
consensus  961 ****************************.****************.********.*****
```

```
MG1655    1021 TRKLREQNSSLPIWGLTANAQANEREKGLSCGMNLCLFKPLTLDVLKTHLSQLHQVAHIA
HS        1021 ............................................................
H10407    1021 .........................................................N..
TW10509   1021 .......HY...V............D...N..............................
EDL933    1021 .............................N.............................V
E2348/69  1021 .............................N..............................
CFT073    1021 .............................N..............................
Nissle    1021 .............................N..............................
UTI89     1021 .............................N..............................
MP1       1021 .............................N..............................
consensus 1021 *******..***.************.***.***************************.*.
```

Histidine – phosphorylation site

```
MG1655    1081 PQYRHLDIEALKNNTANDLQLMQEILMTFQHETHKDLPAAFQALEAGDNRTFHQCIHRIH
HS        1081 ............................................................
H10407    1081 ............................................................
TW10509   1081 .........................................H..................
EDL933    1081 .........................................H..................
E2348/69  1081 .........................................H..................
CFT073    1081 .........................................H..................
Nissle    1081 .........................................H..................
UTI89     1081 .........................................H..................
MP1       1081 .........................................H..................
consensus 1081 *****************************************.******************
```

```
MG1655    1141 GAANILNLQKLINISHQLEITPVSDDSKPEILQLLNSVKEHIAELDQEIAVFCQKND
HS        1141 .........................................................
H10407    1141 .........................................................
TW10509   1141 .............L..................K.....................Q..
EDL933    1141 ......................................................Q.N
E2348/69  1141 .................................................TA...Q..
CFT073    1141 .................................................T....Q..
Nissle    1141 .................................................T....Q..
UTI89     1141 .................................................T....Q..
MP1       1141 .................................................T....Q..
consensus 1141 *************.******************.****************..***.*.
```

S6 Fig. Alignment of EvgS from ten E. coli isolates. EvgS protein sequences from the indicated ten *E. coli* isolates were aligned as described in Materials and methods. (*) indicates a predicted transmembrane domain (www.uniProt.org) that is inconsistent with a homology model based on the ortholog BvgS [11]. The predicted PAS domain is shaded gray, and the green highlights indicate residues identified in previous studies as being involved in EvgS activity [4, 9, 10, 11].
