# Supplementary material for: Natural variation of a sensor kinase controlling a conserved stress response pathway in Escherichia coli
Source: PLoS Genet. 2017 Nov 15;13(11):e1007101. doi: 10.1371/journal.pgen.1007101 (PMC5706723; doi:10.1371/journal.pgen.1007101)
Supplement: S7 Fig — The colors correspond to phylogenetic groups: red–group A, orange–group C, green–group B1, light blue–group D, dark blue–group E, purple–group B2. The phylogenetic groups are also appended to the genome identifiers in the figure. Full-length EvgS sequences were extracted from complete E. coli genomes in NCBI using tblastn (http://blast.ncbi.nlm.nih.gov) with the E. coli K-12 EvgS sequence, and the large number of redundant hits from the genomes of E. coli K-12 derivatives were removed, resulting in 285 EvgS sequences. The tree construction is described in Materials and methods. (PDF) [file pgen.1007101.s013.pdf]

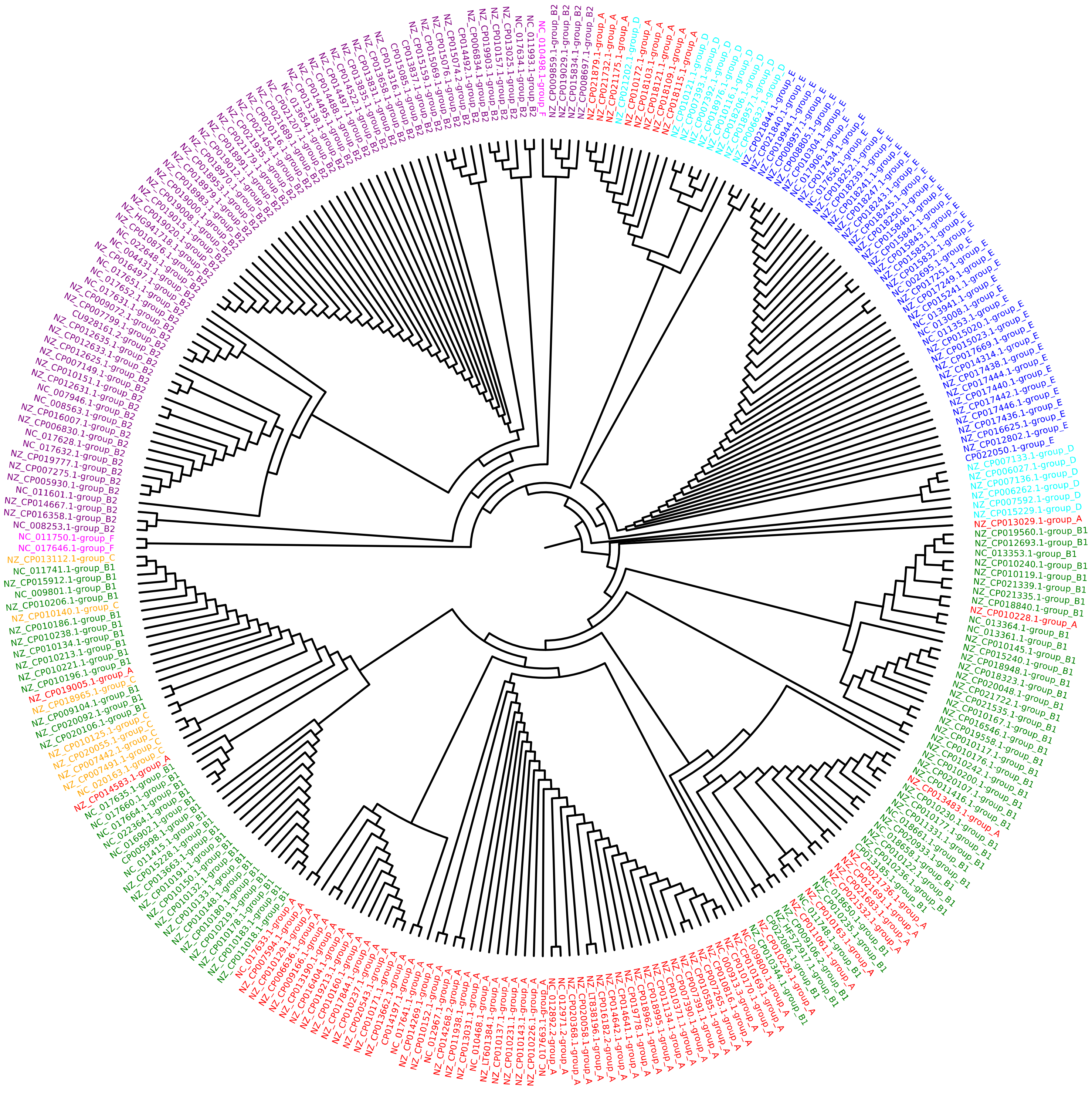

S7 Fig. EvgS phylogeny in E. coli isolates. Dendrogram based on EvgS sequences from 285 *E. coli* genomes. The colors correspond to phylogenetic groups: red – group A, orange – group C, green – group B1, light blue – group D, dark blue – group E, purple – group B2. The phylogenetic groups are also appended to the genome identifiers in the figure. Full-length EvgS sequences were extracted from complete *E. coli* genomes in NCBI using tblastn (http://blast.ncbi.nlm.nih.gov) with the *E. coli* K-12 EvgS sequence, and the large number of redundant hits from the genomes of *E. coli* K-12 derivatives were removed, resulting in 285 EvgS sequences. The tree construction is described in Materials and methods.
