# Supplementary material for: Natural variation of a sensor kinase controlling a conserved stress response pathway in Escherichia coli
Source: PLoS Genet. 2017 Nov 15;13(11):e1007101. doi: 10.1371/journal.pgen.1007101 (PMC5706723; doi:10.1371/journal.pgen.1007101)
Supplement: S8 Fig — The indicated protein sequences from ten E. coli isolates were aligned as described in Materials and methods. Matrix entries indicate the percentage of amino acids that differ for the corresponding pairs of strains. In addition to EvgS and EvgA, matrices are shown for YfdE, a protein encoded by a gene adjacent to evgS, and for two other hybrid histidine kinases, BarA and ArcB. All five matrices use the indicated color range scale. (PDF) [file pgen.1007101.s014.pdf]

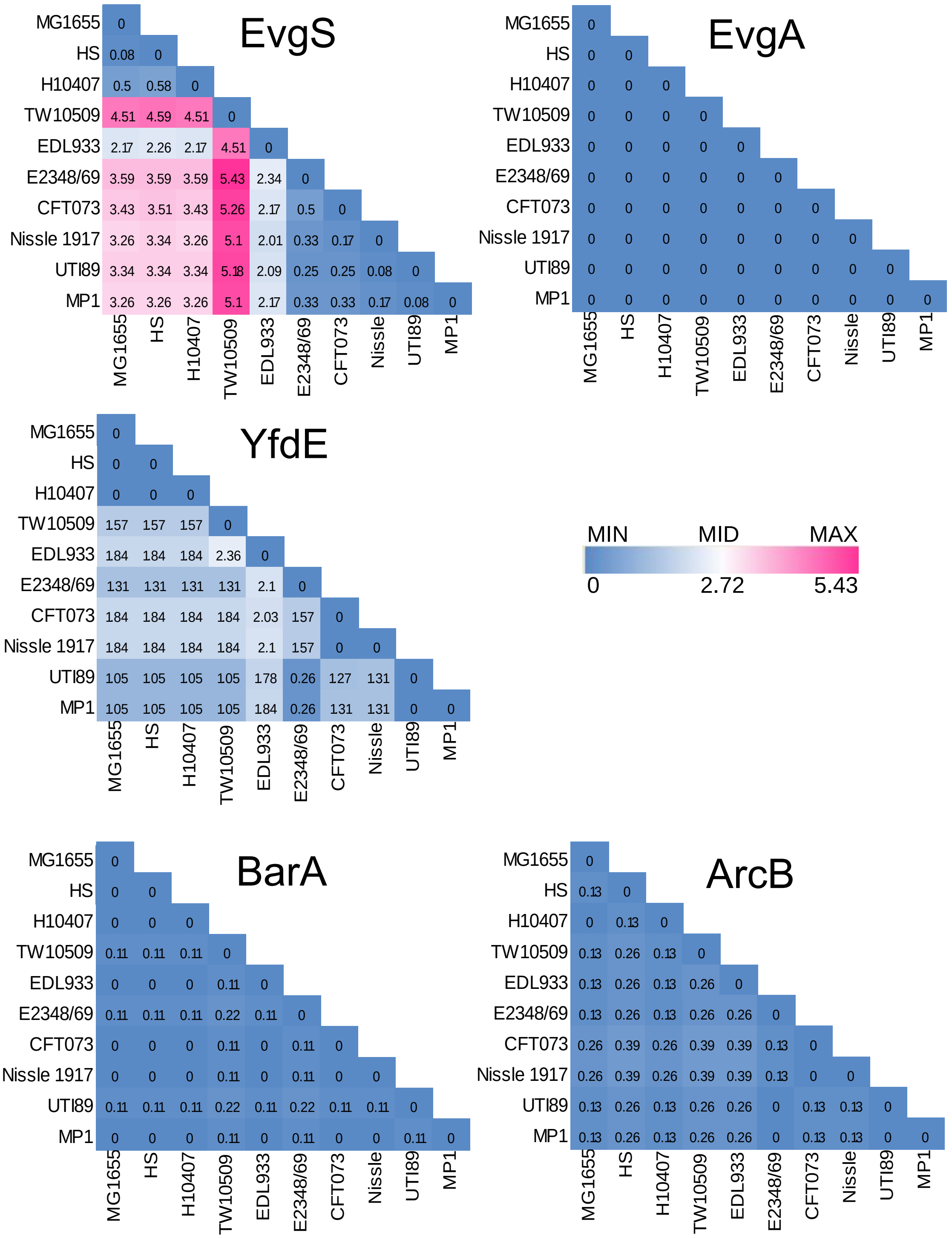

S8 Fig. Protein divergence matrices of ten *E. coli* isolates. The indicated protein sequences from ten *E. coli* isolates were aligned as described in Materials and methods. Matrix entries indicate the percentage of amino acids that differ for the corresponding pairs of strains. In addition to EvgS and EvgA, matrices are shown for YfdE, a protein encoded by a gene adjacent to *evgS*, and for two other hybrid histidine kinases, BarA and ArcB. All five matrices use the indicated color range scale.
