# Supplementary material for: Natural variation of a sensor kinase controlling a conserved stress response pathway in Escherichia coli
Source: PLoS Genet. 2017 Nov 15;13(11):e1007101. doi: 10.1371/journal.pgen.1007101 (PMC5706723; doi:10.1371/journal.pgen.1007101)
Supplement: S9 Fig — A: indicated strains were cultured in minimal medium at pH 5.7 to OD600 ~0.2, and shocked for an hour in LB at pH 2.25 or pH 2.0, or pH 1.75 as described in Materials and methods. Percent survival values are the average of two representative experiments and error bars represent the range. As a reference, the figure includes the values of challenge at pH 2.5 from Fig 5C. B: Correlations between fold induction of the fluorescent reporter PyfdX-yfp from Fig 5B versus percent survival after exposure to pH 2.25, pH 2.0, and pH 1.75 from panel A. The E. coli strains are described in detail in Table 1. Dashed lines represent the limit of detection of the assay. (PDF) [file pgen.1007101.s015.pdf]

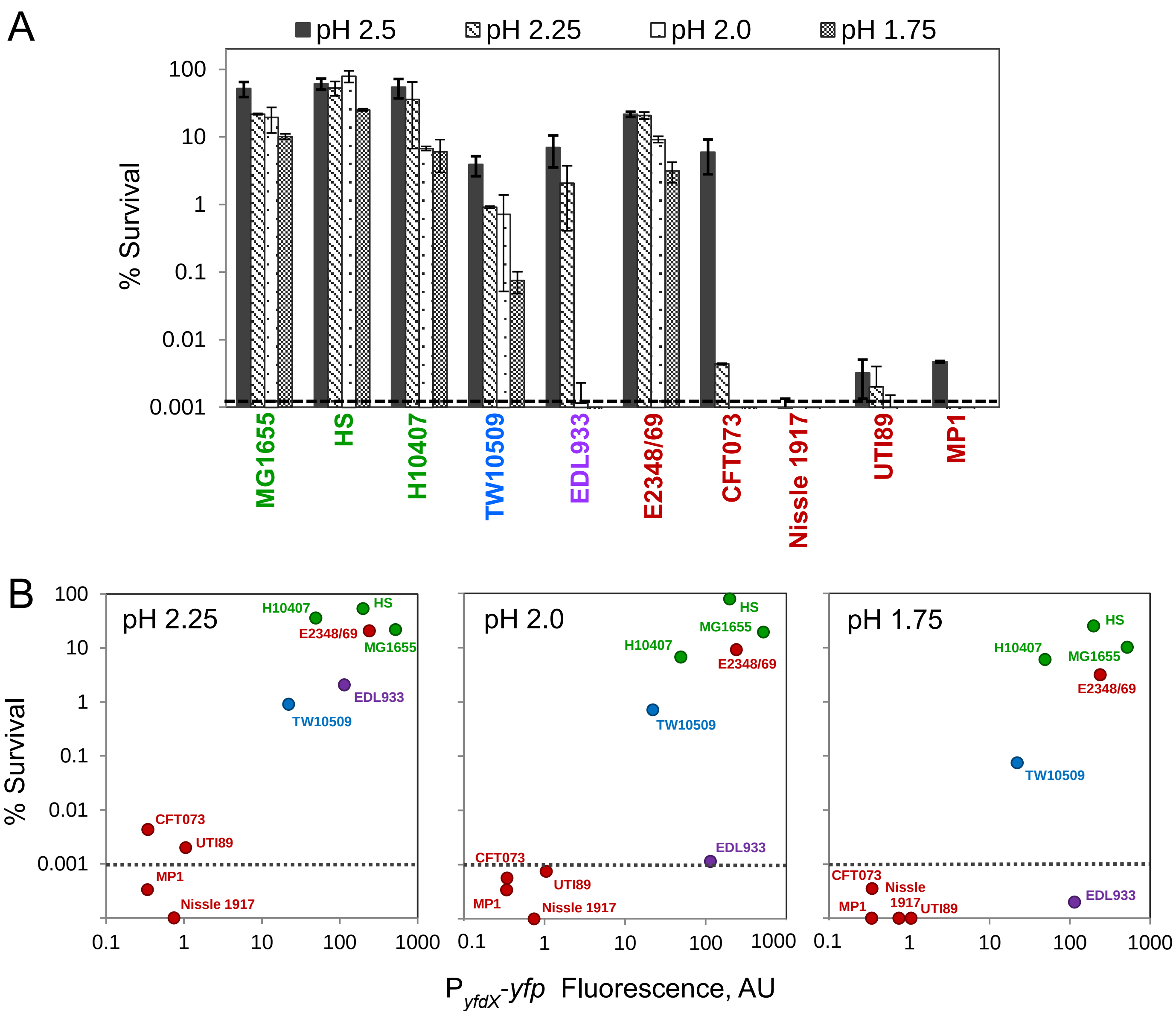

S9 Fig. Sensitivity of selected *E. coli* isolates to acid challenge in a range of pH values. A: indicated strains were cultured in minimal medium at pH5.7 to OD_{600} ~0.2, and shocked for an hour in LB at pH2.25 or pH2.0, or pH1.75 as described in Materials and methods. Percent survival values are the average of two representative experiments and error bars represent the range. As a reference, the figure includes the values of challenge at pH2.5 from Figure 5C. B: Correlations between fold induction of the fluorescent reporter P_{yfdX}-*yfp* from Figure 5B versus percent survival after exposure to pH2.25, pH2.0, and pH1.75 from panel A. The *E. coli* strains are described in detail in Table 1. Dashed lines represent the limit of detection of the assay.
