# Supplementary material for: Natural variation of a sensor kinase controlling a conserved stress response pathway in Escherichia coli
Source: PLoS Genet. 2017 Nov 15;13(11):e1007101. doi: 10.1371/journal.pgen.1007101 (PMC5706723; doi:10.1371/journal.pgen.1007101)
Supplement: S10 Fig — Derivatives of MG1655, Nissle1917 and UTI89 with the PhdeA-yfp reporter (Panel A, MMR175, MMR254 and MMR255) or with the PmgrB-yfp reporter (Panel B, TIM95, MMR272 and MMR273), and carrying the empty vector pSMART or its derivative pevgASMG1655 (pMR78), were cultured in minimal medium at pH 7 and pH 5.7 to OD600~0.2. Fluorescence of the reporter was measured as described in Materials and methods. Fluorescence values are the average from two representative independent experiments. Error bars represent the range. (PDF) [file pgen.1007101.s016.pdf]

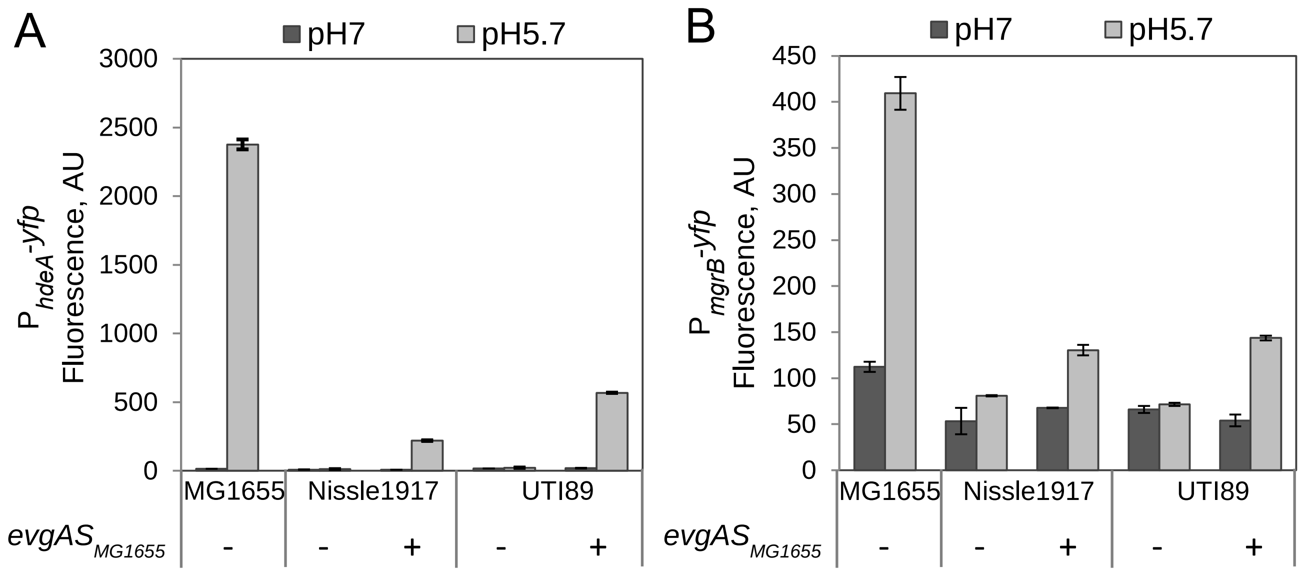

S10 Fig. EvgAS$_{MG1655}$ rescue of the SafA and YdeO pathways in *E. coli* Nissle 1917 and *E. coli* UTI89. Derivatives of MG1655, Nissle1917 and UTI89 with the P$_{hdeA}$-*yfp* reporter (Panel A, MMR175, MMR254 and MMR255) or with the P$_{mgrB}$-*yfp* reporter (Panel B, TIM95, MMR272 and MMR273), and carrying the empty vector pSMART or its derivative p*evgAS*$_{MG1655}$ (pMR78), were cultured in minimal medium at pH7 and pH5.7 to OD$_{600}$~0.2. Fluorescence of the reporter was measured as described in Materials and methods. Fluorescence values are the average from two representative independent experiments. Error bars represent the range.
